# Circulating (Poly)phenol Metabolites: Neuroprotection in a 3D Cell Model of Parkinson's Disease

**DOI:** 10.1002/mnfr.202100959

**Published:** 2022-01-08

**Authors:** Rafael Carecho, Inês Figueira, Ana Paula Terrasso, Joana Godinho‐Pereira, Catarina de Oliveira Sequeira, Sofia Azeredo Pereira, Dragan Milenkovic, Marcel Leist, Catarina Brito, Cláudia Nunes dos Santos

**Affiliations:** ^1^ CEDOC NOVA Medical School Faculdade de Ciências Médicas Universidade NOVA de Lisboa 1150‐082 Lisboa Portugal; ^2^ ITQB Instituto de Tecnologia Química e Biológica António Xavier Universidade Nova de Lisboa 2780‐157 Oeiras Portugal; ^3^ iBET Instituto de Biologia Experimental e Tecnológica 2781–901 Oeiras Portugal; ^4^ INRAE UNH Université Clermont Auvergne 63122 St Genes Champanelle France; ^5^ Department of Nutrition University of California Davis 95616 Davis CA USA; ^6^ In‐vitro Toxicology and Biomedicine University of Konstanz 78457 Constance Germany

**Keywords:** dopaminergic neurons, gene expression, neurodegeneration, preconditioning, transcriptomics

## Abstract

**Scope:**

Diets rich in (poly)phenols have been associated with positive effects on neurodegenerative disorders, such as Parkinson's disease (PD). Several low‐molecular weight (poly)phenol metabolites (LMWPM) are found in the plasma after consumption of (poly)phenol‐rich food. It is expected that LMWPM, upon reaching the brain, may have beneficial effects against both oxidative stress and neuroinflammation, and possibly attenuate cell death mechanisms relate to the loss of dopaminergic neurons in PD.

**Methods and Results:**

This study investigates the neuroprotective potential of two blood‐brain barrier permeant LMWPM, catechol‐*O*‐sulfate (cat‐sulf), and pyrogallol‐*O*‐sulfate (pyr‐sulf), in a human 3D cell model of PD. Neurospheroids were generated from LUHMES neuronal precursor cells and challenged by 1‐methyl‐4‐phenylpyridinium (MPP^+^) to induce neuronal stress. LMWPM pretreatments were differently neuroprotective towards MPP^+^ insult, presenting distinct effects on the neuronal transcriptome. Particularly, cat‐sulf pretreatment appeared to boost counter‐regulatory defense mechanisms (preconditioning). When MPP^+^ is applied, both LMWPM positively modulated glutathione metabolism and heat‐shock response, as also favorably shifting the balance of pro/anti‐apoptotic proteins.

**Conclusions:**

Our findings point to the potential of LMWPM to trigger molecular mechanisms that help dopaminergic neurons to cope with a subsequent toxic insult. They are promising molecules to be further explored in the context of preventing and attenuating parkinsonian neurodegeneration.

## Graphical abstract

Neuroprotective effects of LMWPM pretreatment in LUHMES neurospheroids are challenged by MPP^+^. LMWPM induces differential modulation of gene expression that overall promotes an adaptative response to better cope with MPP^+^ insult. Particularly, cat‐sulf, by positively modulating glutathione metabolism, apoptotic proteins balance, and heat‐shock response, it promotes the increase of cell viability and ATP levels restoration.

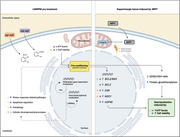

## 1 Introduction

Parkinson's disease (PD) is the second most common neurodegenerative disorder and the first motor debilitating degenerative disease.^[^ ^1^, ^2^ ^]^ PD is a chronic pathology that affects the brain due to the loss of dopaminergic neurons in the *substantia nigra pars compacta* and the presence of protein aggregates containing α‐synuclein.^[^ ^3^ ^]^ PD symptoms result from a decrease in dopamine levels which might be caused by a disturbance in dopamine exchange system.^[^ ^3^ ^]^

Dopaminergic neurons in PD display defects in mitochondrial function, increased oxidative stress, and neuroinflammation.^[^ ^4^ ^]^ Through the past years, it has been shown that oxidative stress is central to the pathology of this disease.^[^ ^5^ ^]^ Moreover, the *substantia nigra* of PD patients shows increased levels of oxidized lipids, proteins, and DNA, together with a decrease in reduced glutathione (GSH) levels, the major antioxidant defense in the cells.^[^ ^6^ ^]^ In PD, a main source of free radicals stems from alterations in complex I of the mitochondrial respiratory chain, which compromises proper mitochondrial functioning and ATP production, leading to cell death.^[^ ^7^ ^]^ MPTP (1‐methyl‐4‐phenyl‐1,2,3,6‐tetrahydropyridine), the prodrug of the neurotoxin MPP^+^ (1‐methyl‐4‐phenylpyridinium), has been widely used to study the disease pathophysiology once it leads to outcomes similar to PD.^[^ ^8^ ^]^ In fact, dopaminergic neurons are selectively vulnerable due to dopamine transporter (DAT) high‐affinity for MPP^+^, which subsequentially impairs electron transport chain.^[^ ^9^ ^]^

The prevention and treatment of PD complex pathobiology urgently need novel strategies targeted to multiple genes and proteins. (Poly)phenols, which can be found mainly in fruits and vegetables, exhibit a remarkable multipotent ability to modulate several mechanisms common to all neurodegenerative disorder like oxidative stress, metal toxicity, inflammation and immune response, apoptosis, signal transduction, ion channels, among others (reviewed in Bhullar and Rupasinghe^[^ ^10^ ^]^). For instance, (poly)phenols like resveratrol,^[^ ^11^ ^]^ epigallocatechin gallate,^[^ ^12^ ^]^ and quercetin,^[^ ^13^ ^]^ despite their poor absorption and limited brain availability, were shown to modulate PD‐associated progressive dopaminergic degeneration, mitochondrial dysfunction, apoptosis, protein aggregation, and neuroinflammation. However, the mechanism of action of the low molecular weight (poly)phenol metabolites (LMWPM), which are the most abundant and simple metabolites of (poly)phenols found in circulation, is poorly understood. Such circulating LMWPM can exert genomic modifications in brain cells,^[^ ^14^ ^]^ presenting a strategy for disease prevention with potential to be explored. Simple phenolic sulfates, in particular colonic‐derived phase II metabolites of dietary (poly)phenols were identified in biological samples of human volunteers which took a berries mixture,^[^ ^15^, ^16^ ^]^ cranberries,^[^ ^17^ ^]^ mango,^[^ ^18^ ^]^ or black tea.^[^ ^19^ ^]^ These sulfates, when tested in circulating concentrations (5 µM), were shown to be significantly transported across an in vitro model of the blood‐brain barrier (BBB),^[^ ^20^ ^]^ holding great promise as possible brain‐targeted compounds. In fact, these metabolites, in particular catechol‐*O*‐sulfate (cat‐sulf) and pyrogallol‐*O*‐sulfate (pyr‐sulf), presented strong neuroprotective and anti‐neuroinflammatory activity against common neurodegeneration hallmarks in vitro.^[^ ^20^ ^]^ However, their role in the context of PD is still unexplored.

The present study aims to investigate the protective potential of selected human bioavailable LMWPM, simple phenolic sulfates, derived from the human metabolism of dietary (poly)phenols.^[^ ^15^ ^]^ A human 3D neural cell model generated from immortalized human dopaminergic neuronal precursor cells, the Lund Human Mesencephalic (LUHMES) cell line, was used. LUHMES were differentiated into neurospheroids containing post‐mitotic neurons with enrichment in dopaminergic neurons, in an agitation‐based culture system.^[^ ^9^, ^21^ ^]^ For induction of a PD‐like phenotype, differentiated LUHMES neurospheroids were treated with the dopaminergic neurotoxicant MPP^+^. The 3D cell model of PD was then employed to assess the effects of physiological concentrations of both LMWPM cat‐sulf and pyr‐sulf. LMWPM revealed to be differently neuroprotective towards the dopaminergic lesion applied. Moreover, both were able to modulate several genes mainly involved in stress response, and have also shown ability to interfere with glutathione metabolism, comprising promising new molecules to be further explored in the scope of PD.

## 2 Results

### 2.1 Circulating LMWPM Are Neuroprotective Towards MPP^+^

A 3D cell model of PD has been implemented using dopaminergic differentiated LUHMES neurospheroids and an MPP^+^‐induced lesion. Neurospheroids are obtained until 14 days differentiation and are enriched in post‐mitotic neurons (Figure S1, Supporting Information), as indicated by the increased mRNA and protein levels of major neuronal (e.g., bIII‐tubulin, synaptophysin) and dopaminergic (e.g., tyrosine hydroxylase (TH), DAT) markers, peaking at 7 days of differentiation, with concomitant decrease in early neuroepithelial progenitor markers (e.g., Nestin) (Figure S2, Supporting Information). These 7 days‐neurospheroids were enriched in TH‐positive neurons (Figure S2, Supporting Information). MPP^+^ lesion reduced cell viability in a dose‐dependent manner, with an IC_50_ of 5 µM. A downregulation in the dopaminergic TH gene expression was observed along lesion time, with no significant alterations in bIII‐tubulin and DAT gene expression, nor βIII‐tubulin and TH protein levels (Figure S3, Supporting Information).

**Figure 1:**
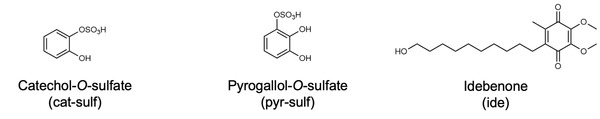
Chemical structure of the LMWPM, cat‐sulf and pyr‐sulf, as well as the drug ide.

**Figure 2:**
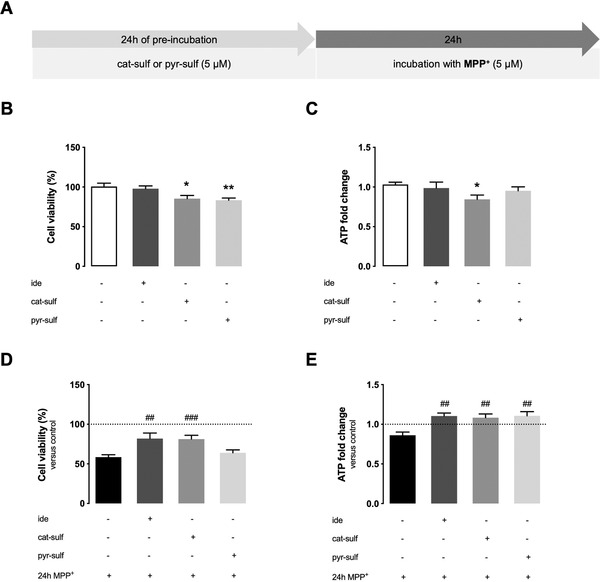
Neuroprotective potential of LMWPM against MPP+ lesion in LUHMES neurospheroids. A) Standard experimental setup for differentiated neurospheroids. They were preincubated for 24 h with 5 µM of each LMWPM, cat‐sulf or pyr‐sulf or with 200 nM ide. LUHMES neurospheroids were then incubated for 24 h with new medium containing or not 5 µM of MPP+. Cell viability was assessed by Presto blue assay after 24 h of injury and ATP fold change was assessed by CellTiter Glo Assay after 48 h of injury, both expressed relatively to control condition. LMWPM (cat‐sulf, pyr‐sulf) and ide toxicity in LUHMES neurospheroids after 24 h of incubation in terms of B) cell viability, and C) ATP levels. Neuroprotection evidenced in terms of D) cell viability or E) ATP fold change relative to control without MPP^+^ (indicated by dashed line). Statistical differences are denoted as **p < 0.01 and *p < 0.05 versus control and denoted as ###*p* < 0.001 and ##*p* < 0.01 versus MPP+ insult obtained by one‐way ANOVA analysis with Tukey's post multiple comparison test. Data are mean ± SEM of three independent cultures.

LUHMES 7 days‐neurospheroids were incubated for 24 h with physiologic‐circulating concentrations (5 µM) of two LMWPM: cat‐sulf and pyr‐sulf.^[^ ^15^, ^16^, ^20^ ^]^ In addition, the coenzyme Q10 analogue of ide (**Figure** **1**), a known antioxidant drug,^[^ ^22^ ^]^ was used as positive control. The pretreated cells were exposed to the known dopaminergic toxicant MPP^+^ (5 µM) for 24 h (**Figure** **2**A).

Cell viability was assessed by the resazurin reduction assay (readout related to mitochondrial/cell metabolic activity) and by measurement of the overall ATP content (measure of cell integrity and of bioenergetics functions). We observed that cat‐sulf per se caused a slight decrease of both endpoints, while pyr‐sulf alone induced a decrease in resazurin reduction, but not in ATP levels (Figure 2B,C). Despite the apparently dampening effect on cell viability endpoints by both LMWPM, they differently protected the neurons from the large damage triggered by MPP^+^: preincubation either with ide or cat‐sulf, but not pyr‐sulf, mitigated the effect on resazurin reduction (Figure 2D), while both cat‐sulf and pyr‐sulf, as well as ide, were able to maintain normal ATP levels in the presence of MPP^+^ (Figure 2E).

Overall, these data suggest that the two LMWPM tested, besides induced a differential neuroprotection, they may trigger a preconditioning cellular response that may help the cells to cope later with the MPP^+^ lesion.

### 2.2 Circulating LMWPM Differentially Modulate Gene Expression

**Figure 3:**
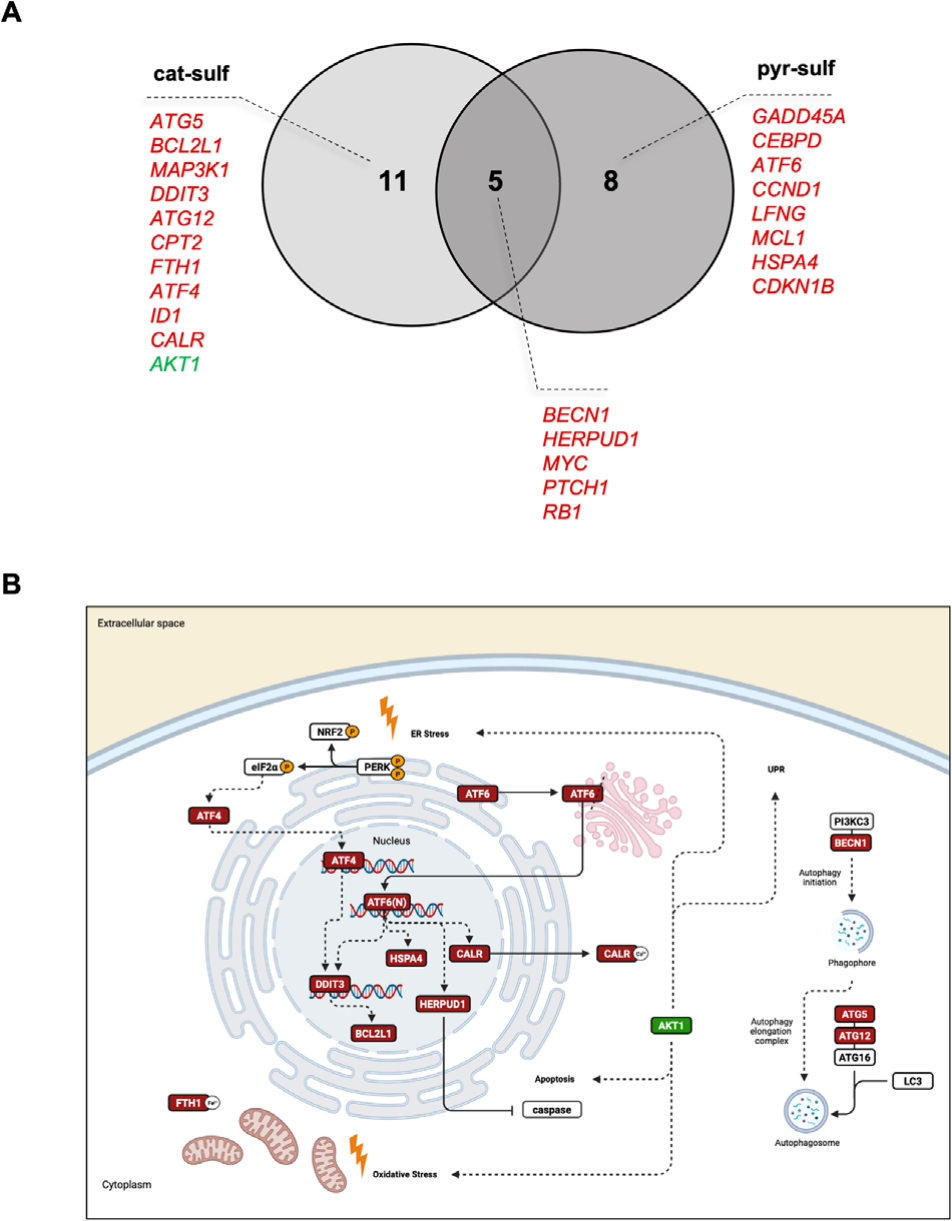
Circulating LMWPM differentially modulate gene expression. A) Venn diagram based on gene transcription analysis after cat‐sulf or pyr‐sulf treatment. Statistical significance was assessed by Student's *t*‐test, *p*‐value <0.05, different from control. B) Schematic representation illustrating points of contact between significantly different expressed genes associated to oxidative stress pathway targets (*FTH1*), apoptosis targets (*AKT1*, *BCL2L1*), autophagy targets (*ATG5*, *ATG12*, *BECN1*) and unfolded protein response (UPR) targets (*ATF4*, *ATF6*, *DDIT3*, *CALR*, *HSPA4*, *HERPUD1*). Adapted from BioRender.com. Upregulated genes are depicted in red and downregulated genes are depicted in green. Descriptive of genes function indicated in Table S2, Supporting Information, for each gene symbol.

To evaluate the mechanism and signaling pathways underlying this protective response, a PCR array was performed to evaluate key players in the top signal transduction pathways affected during the preincubation of LUHMES neurospheroids with cat‐sulf and pyr‐sulf alone, before the MPP^+^ insult. Of the total 93 target genes assessed, 66 were amplified. Both cat‐sulf and pyr‐sulf treatment induced changes on gene transcription, with fold changes ranging from –4.63 to 13.7, for cat‐sulf, and –3.12 to 7.08, for pyr‐sulf. Sixteen genes were modulated by cat‐sulf and 13 by pyr‐sulf, with only five in common (**Figure** **3**A). From the total 24 differentially expressed genes, all of them were upregulated excepting *AKT1*, specifically downregulated by cat‐sulf. All these differentially expressed genes appeared associated with neurodegenerative diseases and, in particular, with PD, as expected (Figure S4, Supporting Information). Interestingly, most of the genes that were changed by the metabolites comprise targets of oxidative stress pathway (*FTH1*), apoptosis (*AKT1*, *BCL2L1*), autophagy (*ATG5*, *ATG12*, *BECN1*) or unfolded protein response (UPR) (*ATF4*, *ATF6*, *DDIT3*, *CALR*, *HSPA4*, *HERPUD1*), sharing common functions in the cell (Figure 3B).

#### 2.2.1 Pathway Enrichment Analysis Identified Distinct Regulation Processes Associated with Each LMWPM Metabolites‐Induced Expressed Genes

**Figure 4:**
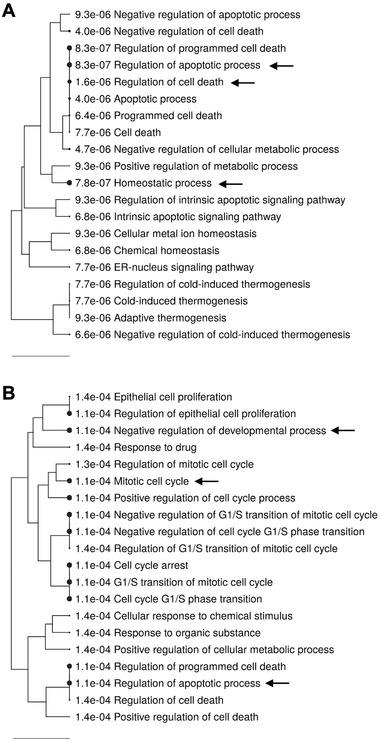
Gene ontology (GO) enrichment analysis of genes differently expressed in LUHMES cells treated with LMWPM, using ShinyGO online tool. Hierarchical clustering tree showing the biological function of the differentially expressed genes induced by both A) cat‐sulf and B) pyr‐sulf. Pathways are clustered considering the number of shared genes. Bigger dots represent more significant *p*‐values (cut‐off 0.05). Black arrows indicate the most significant and relevant pathways.

Based on the gene ontology analysis of the differentially expressed genes, a hierarchical clustering tree was built, revealing the pathways with more shared genes clustered together (**Figure** **4**): for cat‐sulf, apoptosis regulation, cell death, and cell homeostasis were the most significant (Figure 4A), while for pyr‐sulf were cell death and apoptosis regulation, cell replication, as well as cellular developmental processes (Figure 4B).

**Figure 5:**
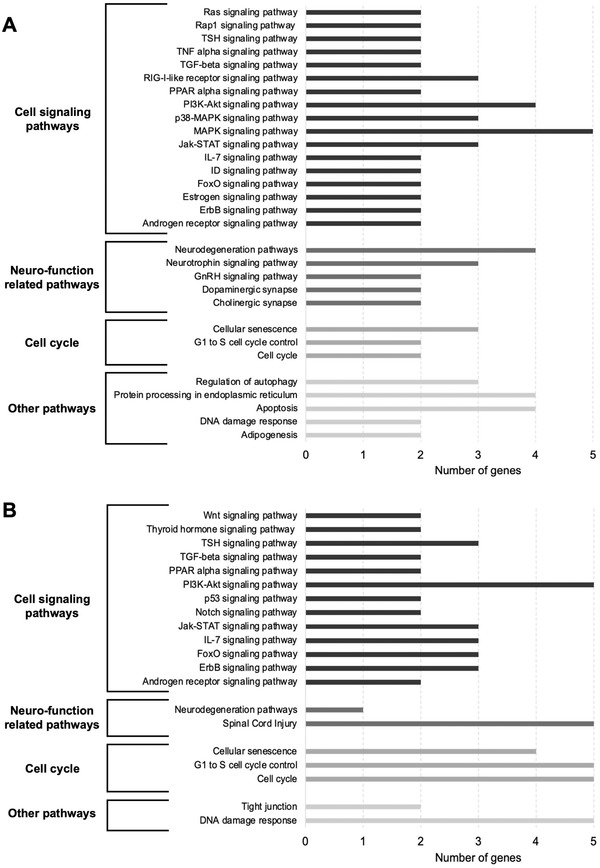
Enriched cellular pathways of protein‐coding genes differently expressed in LUHMES cells treated with LMWPM, using GeneTRial2 software. Histogram showing the number of genes mapped to the enriched pathways grouped in cell signaling pathways, neurofunction related pathways, cell cycle pathways, or other pathways for both A) cat‐sulf and B) pyr‐sulf.

Cellular pathway analysis highlighted processes related to neuronal function, cell cycle, or cell signaling, which were affected by both metabolites (**Figure** **5**). Among the pathways identified which were common for the two LMWPM were cellular senescence, IL‐7, Jak‐STAT, PI3K‐Akt, and PPAR alpha signaling pathways. Other pathways were identified as specific for cat‐sulf or pyr‐sulf: following treatment with cat‐sulf, genes regulating neurotrophic signaling pathway, but also apoptosis, protein processing in ER or MAPK signaling pathway emerged (Figure 5A), whereas pathways specific to pyr‐sulf comprised p53 network, Notch signaling, tight junction, or Wnt signaling pathways (Figure 5B).

**Figure 6:**
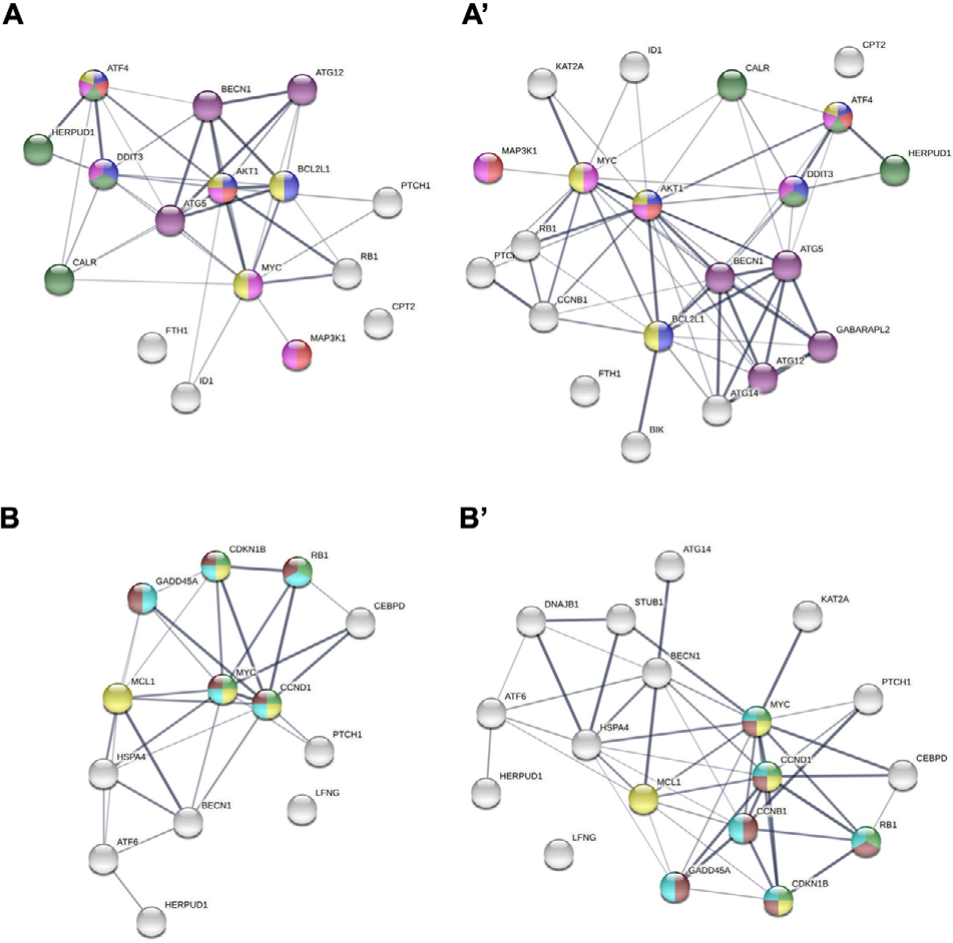
Protein–protein interaction networks functional enrichment analysis of proteins coded by the genes differently expressed in LUHMES cells treated with LMWPM, using the database STRING. Network of protein associations coded by the genes affected by A) cat‐sulf and or B) pyr‐sulf and interactions between those with other proteins whose activity could be indirectly affected by gene expression modulation of cat‐sulf A**’**) or pyr‐sulf B’). Network nodes represent proteins connected by lines as thicker as the strength of data support. Colored nodes represent proteins associated to some of the enriched pathways illustrated in Figure 5. For cat‐sulf are represented MAPK signaling pathway (rose node), PI3K‐Akt signaling pathway (yellow node), protein processing in ER (green node), apoptosis (blue node), autophagy (purple node), and neurotrophin signaling pathway (red node). For pyr‐sulf is represented PI3K‐Akt signaling pathway (yellow node), DNA damage response (light blue), G1/S transition of mitotic cell cycle (light green), and cell cycle (brown).

PPI analysis allowed us to identify interactions between proteins coded by the genes identified as differentially expressed (**Figure** **6**A,B). We were able to group protein nodes by pathways involvement, each one represented by a different color, highlighting the enriched cellular pathways with highest number of genes involved.

Also, interactions between those proteins with other proteins whose activity could be indirectly affected by gene expression modulation are presented in Figure 6A’,B’. For cat‐sulf, AKT1 seems to serve as a central hub with the highest number of interactions between proteins. Whereas the protein network associated with cat‐sulf indicates that PPI is involved in apoptosis and stress response, those associated to pyr‐sulf spreads in proteins with a role in cell cycle. Taken together, these analyses allowed us to identify cellular functions in which genes having expression modulated by the metabolites are involved in and can impact cell survival and neuronal cell functions.

#### 2.2.2 Transcription Factors and miRNA Enrichment Analysis Identified Different Potential Players Involved in the Regulation of Gene Expression by Each LMWPM

**Figure 7:**
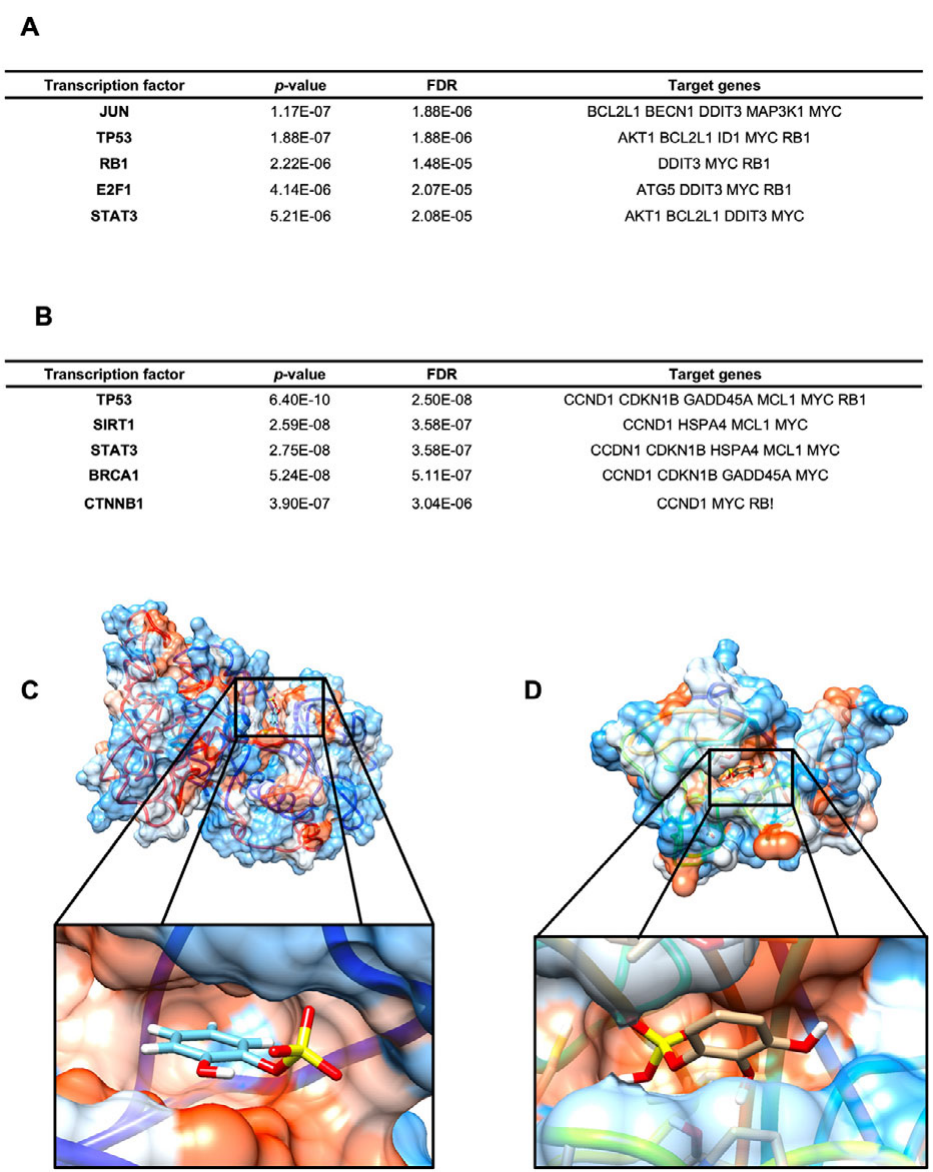
TFs enrichment analysis highlighting TFs potentially involved in the gene expression modifications induced by both LMWPM. List of top five potential TFs whose activity could be affected by the A) cat‐sulf and B) pyr‐sulf, using TTRUST database. Docking analysis by using Mcule molecular modeling tool, for C) cat‐sulf and its top TF JUN and D) pyr‐sulf and its respective top TF TP53.

The next step of our bioinformatic analyses was to identify potential TFs which activity could be regulated by the two LMWPM studied and explain the gene expression modulation observed. This analysis revealed that the top five, among the most probable ones for cat‐sulf treatment (lower *p*‐value), were JUN, TP53, RB1, E2F1, and STAT3 (**Figure** **7**A), having as target genes *BCL2L1*, *MAP3K1*, *AKT1*, *DDIT3*, *MYC*, *RB1*. In silico docking analysis for JUN and cat‐sulf suggests a favorable binding with minimal energy of −6.25 kcal mol^–1^ (Figure 7C). For pyr‐sulf, the potential TFs identified were TP53, SIRT1, STAT3, BRCA1, and CTNNB1 (Figure 7B). Comparison between the top five most probable TFs identified two of them as common for cat‐sulf and pyr‐sulf, namely TP53 and STAT3.

**Figure 8:**
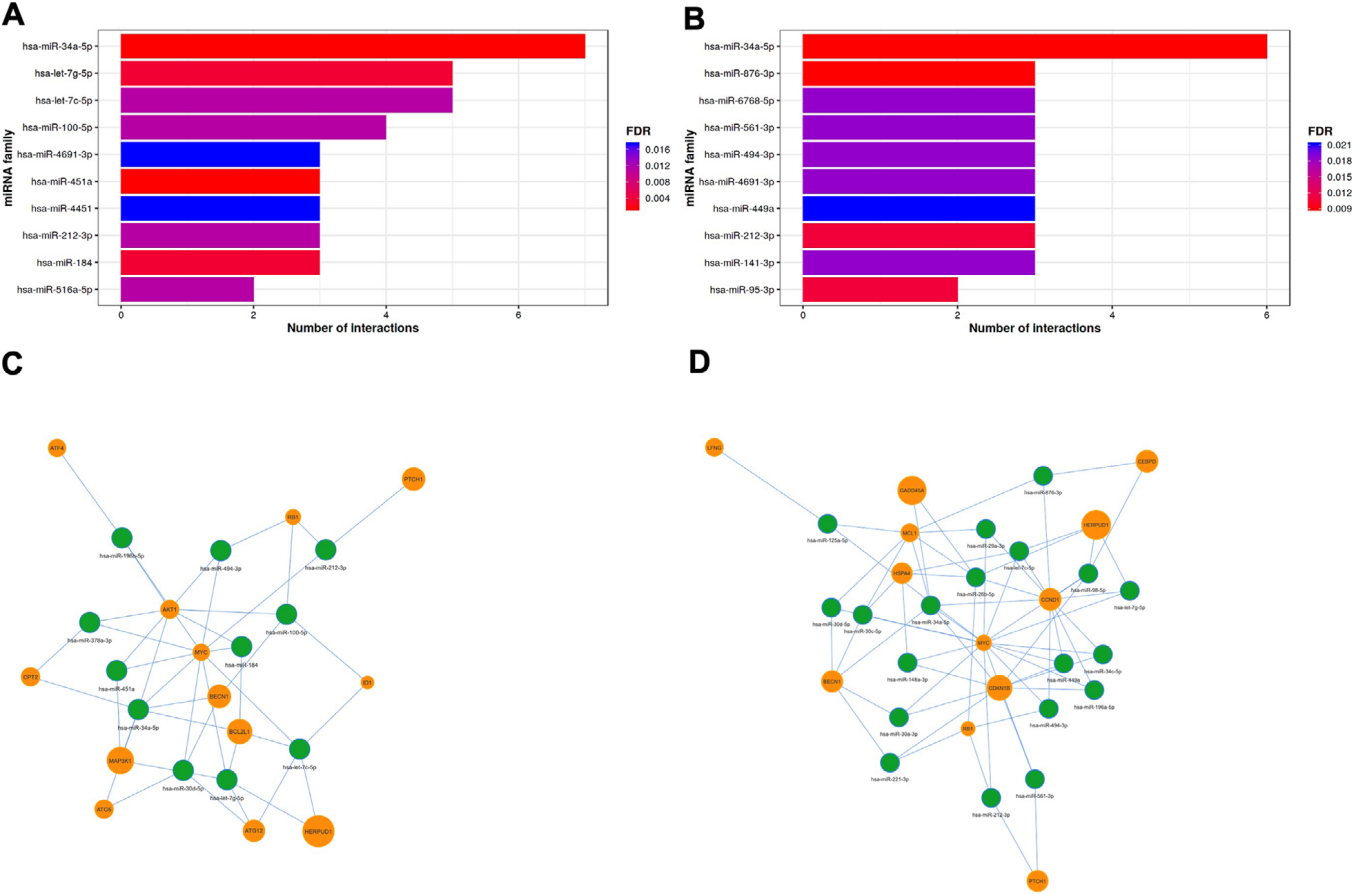
Enrichment analysis of miRNAs potentially involved in post‐transcriptional regulation of the identified differentially expressed genes in LUHMES cells treated with LMWPM, based on prediction database miRTarBase, using MIENTURNET online tool. Histogram showing the number of possible interactions between top 10 miRNAs and differentially expressed genes by A) cat‐sulf and B) pyr‐sulf. The following settings were applied: minimum interactions of miRNA‐gene—3; adjusted *p*‐value (FDR)—0.05. Network of miRNA–gene interactions for both C) cat‐sulf and D) pyr‐sulf. By default, miRNAs are represented as green circles, while target nodes (genes) are orange circles.

Besides TFs, genes can also be regulated at post‐transcriptional level, including via miRNAs. Among the miRNA identified as having higher number of target interactions and potentially involved in gene regulation following exposure to LMWPM, miR‐34a‐5p was the only common to both metabolites. It was also the one with higher number of interactions, with seven predicted interactions for cat‐sulf (**Figure** **8**A) and six for pyr‐sulf (Figure 8B). The interactions between miRNAs and differentially expressed genes were also identified for cat‐sulf and pyr‐sulf (Figure 8C,D, respectively), showing that these genes and miRNAs are interconnected and form a network that infers about miRNA regulation on the target genes.

Collectively, we observed that both cat‐sulf and pyr‐sulf treatment trigger few common genes but affects mostly different genes (Figure 3), pointing out to different pathway modulation (Figure 5). Consequently, this implicates that, before the challenge with MPP^+^, cells presented unequal metabolic alterations.

### 2.3 Circulating LMWPM Induce Changes in Glutathione Profile After MPP^+^ Lesion

**Figure 9:**
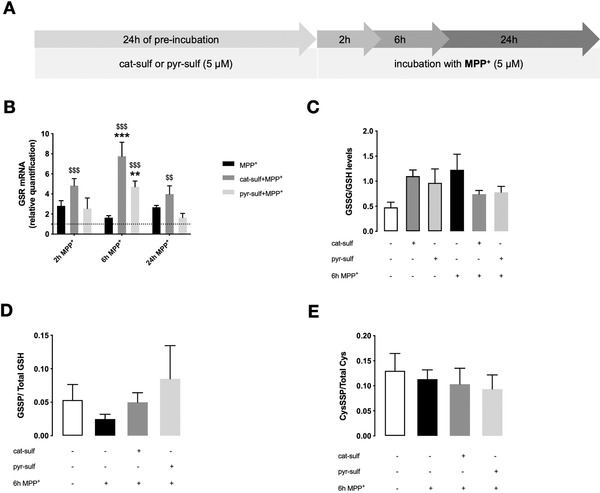
Effects of LMWPM on glutathione dynamics in LUHMES neurospheroids treated with MPP^+^. A) Standard experimental setup for differentiated neurospheroids. They were preincubated for 24 h with 5 µM of each LMW (poly)phenol metabolite, cat‐sulf, or pyr‐sulf. LUHMES neurospheroids were then incubated up to 24 h (2, 6, and 24 h) with new medium containing 5 µM of MPP^+^. B) Relative mRNA expression by RT‐qPCR of GSR upon LMWPM preincubation at 2, 6, or 24 h of MPP^+^. C) GSSG/GSH ratio was calculated as GSSG/(GSH+2*GSSG) upon LMWPM preincubation after 6 h of MPP^+^. D) Ratio between S‐glutathionylated protein (GSSP) levels and total free available GSH, and E) ratio between total S‐cysteinylated proteins (CysSSP) levels and total free available cysteine, both upon LMWPM preincubation after 6 h of MPP^+^. Statistical differences are denoted as ***p < 0.001 relative to respective time point of MPP^+^ insult and ^$$^ *p* < 0.01, ^$$$^ *p* < 0.001 relative to respective control group with no MPP^+^ by two‐way ANOVA analysis with Bonferroni's post multiple comparison test. Data are mean ± SEM of three independent cultures.

By inhibiting complex I in mitochondria, MPP^+^ promotes the increase of ROS levels, leading to oxidative stress. In this scenario, we may find an MPP^+^‐impaired glutathione (GSH) system, which is a critical line of defense against oxidative stress in the brain.^[^ ^6^ ^]^ We addressed glutathione dynamics after MPP^+^ insult, according to the experimental setup presented in **Figure** **9**A.

From the mRNA levels of glutathione reductase (*GSR*), we observed that cat‐sulf preincubation was able to significantly increase its expression after MPP^+^ lesion compared with MPP^+^ alone, reaching a maximum of 8‐fold at 6 h after MPP^+^ lesion (Figure 9B). This induction was observed from 2 to 24 h of MPP^+^ lesion for cat‐sulf and for pyr‐sulf was only detected at 6 h of lesion. At that time, preincubation with both metabolites tends to reduce the ratio GSSG/GSH, while the metabolites per se tend to increase GSSG/GSH ratio, mainly due to a decrease in GSH levels while GSSG remained unchanged. In fact, catechol rings have been described to oxidize and react with GSH,^[^ ^23^ ^]^ a possible justification for such decrease. Nevertheless, the metabolites were still able to counteract the effect caused by MPP^+^, (Figure 9C) that may reflect the GSR increase observed. Moreover, LMWPM preincubation also led to a slight increase in S‐glutathionylated (GSSP) proteins levels, whereas there were no significant changes in proteins cysteinylation (Figure 9D,E).

These results suggest the ability of LMWPM to maintain a GSH pool in the cells that may be crucial for fighting against free radicals produced in response to MPP+ lesion.

### 2.4 Circulating LMWPM Neuroprotection Acts by Transcriptional Modulation of Apoptosis and Stress Response Players after MPP^+^ Lesion

**Figure 10:**
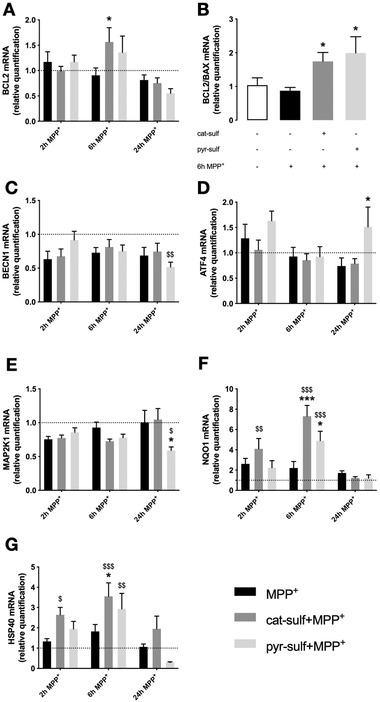
Circulating LMWPM neuroprotective potential through genes modulation in LUHMES neurospheroids treated with MPP^+^. Relative mRNA expression by RT‐qPCR of apoptosis‐related genes A) *BLC2* and B) *BCL2/BAX* ratio. Relative mRNA expression of stress response‐related genes C) *BECN1*, D) *ATF4*, E) *MAP2K1*, F) *NQO1*, and **G**) *HSP40*. Statistical differences are denoted as *p < 0.05, ***p < 0.001 relative to respective time point of MPP^+^ insult and ^$^ *p* < 0.05, ^$$^ *p* < 0.01, ^$$$^ *p* < 0.001 relative to respective control group with no MPP^+^ by two‐way ANOVA analysis with Bonferroni's post multiple comparison test. Data are mean ± SEM of three independent cultures.

To address whether the unequal gene expression induced by each metabolite is responsible for modulating LUHMES cells to face differently MPP^+^ lesion, mRNA levels of genes involved in cellular stress‐related pathways, such as, apoptosis regulation, stress response, and autophagy were considered (**Figure** **10**).

We observed that mRNA levels of major apoptosis players, the anti‐apoptotic *BCL2*, and the pro‐apoptotic *BAX*, were significantly modulated, after 6 h of MPP^+^ lesion. The preincubation with cat‐sulf led to an increase in *BCL2* mRNA levels comparing with 6 h of MPP^+^ alone; however, after 24 h of lesion, cat‐sulf preincubation was no longer able to modulate *BCL2* expression (Figure 10A). Moreover, preincubation with pyr‐sulf did not induce changes in *BCL2* mRNA levels (Figure 10A). Nevertheless, the balance between pro‐ and anti‐apoptotic players can determine the cellular fate and, indeed, *BCL2*/*BAX* ratio reflected the counteraction of both LMWPM towards MPP^+^ treatment, being significantly increased at 6 h of lesion (Figure 10B). The fact we cannot see an increase in *BCL2* promoted by pyr‐sulf, may suggest that its neuroprotection is not via *BAX* expression modulation.

Concerning cellular processes related to stress response and autophagy, *BECN1*, *ATF4*, *MAP2K1*, *NQO1*, and *HSP40* mRNA levels were also evaluated. Pretreatment with pyr‐sulf led to a decrease of *BECN1* at 24 h of MPP^+^ comparing with control group with no metabolites or MPP^+^ (Figure 10C). Pretreatment with cat‐sulf did not alter *BECN1* expression (Figure 10C). ATF4 levels decreased with lesion time, being this trend only counteracted by pyr‐sulf at 24 h of MPP^+^ (Figure 10D). Moreover, pyr‐sulf induced a decrease in *MAP2K1* after 24 h of lesion (Figure 10E). Interestingly, pretreatment with both cat‐sulf and pyr‐sulf before 6 h of MPP^+^ incubation was able to change *NQO1* and *HSP40* expression by increasing its levels comparing with both respective control and MPP^+^ groups with no metabolites pretreatment (Figure 10F,G).

These results strongly suggest that both LMWPM play multiple roles in cellular adaptation to stress, by differently modulating target genes mainly involved in apoptosis and stress response processes.

## 3 Discussion

(Poly)phenol‐rich foods, like fruits and vegetables, have been described to present brain health benefits with positive impact in neurodegeneration.^[^ ^24^, ^25^ ^]^ Despite the low bioavailability and reduced brain accessibility of parent compounds, they seem to originate some common breakdown metabolites, LMWPM, which are in fact those that can be found in circulation and excreted in urine of human volunteers.^[^ ^15^, ^16^, ^17^, ^18^, ^19^ ^]^ These circulating LMWPM comprise, among others, simple phenolic sulfates.^[^ ^15^ ^]^ Two of the most abundant detected after the consumption of a berry mixture puree were cat‐sulf and pyr‐sulf, in which some volunteers reach concentrations as high as 20 µM.^[^ ^15^ ^]^ As such, these bioavailable LMWPM can constitute true effectors in cellular and molecular mechanisms, contributing for the beneficial effects reported in several neurodegenerative diseases. Indeed, some metabolites were already shown to accumulate in the brain, being able to prevent Ab peptides aggregation.^[^ ^26^ ^]^ However, there is still very scarce information on the and bioactivity and bioavailability of the LMWPM, namely in the context of PD. Previously, we demonstrated that both cat‐sulf and pyr‐sulf can be transported across BBB endothelium at physiological concentrations and are neuroprotective in different cell models with increasing complexity,^[^ ^20^ ^]^ despite their potential towards PD was never reported before.

Differentiated LUHMES neurospheroids emerged as a valuable tool for the assessment of LMWPM potential. In this work, we investigated the LMWPM potential against one of the most important hallmarks of PD, which is the death of dopaminergic neurons, here recapitulated by a dopaminergic lesion caused by MPP^+^.^[^ ^27^ ^]^ The 3D model offers physiological advantages compared with 2D cultures, mimicking better the brain environment complexity, presenting cell–cell interactions more closely related to what is observed in vivo.^[^ ^28^ ^]^ Moreover, differentiated LUHMES neurospheroids were enriched in dopaminergic neurons, which expressed DAT, TH, and βIII‐tubulin. Some glial fibrillary acidic protein‐positive cells were also observed at 7 days of differentiation, which can suggest that, in a 3D microenvironment, LUHMES cells may be able to be differentiated into glial lineages. Importantly, a consistent differentiated phenotype was obtained after 7 days of differentiation in 3D, resulting in the irreversible conversion of LUHMES neuronal precursors into a dopaminergic‐enriched neuronal population, comparable with the protocol described by Smirnova and colleagues, using gyratory shaking technique for 21 days of differentiation.^[^ ^27^ ^]^

LUHMES neurospheroids responded to the MPP^+^ lesion, as reported before,^[^ ^9^, ^27^ ^]^ comprising a disease‐relevant cell model to study the potential of LMWPM towards a dopaminergic lesion with significance to PD. Also, although both cat‐sulf and pyr‐sulf alone presented a slight toxicity in LUHMES neurospheroids, we confirmed the neuroprotective effects of cat‐sulf pretreatment after MPP^+^ lesion, by mitigating the loss of cell viability and restoring ATP levels. Despite pyr‐sulf appeared to be capable to recover ATP levels, it was not able to prevent the loss of cell viability. Such observation suggests that LMWPM, in particular, cat‐sulf may preconditioning the cells to stronger insults. In turn, we may hypothesize that the preincubation with each metabolite may trigger different molecular players, or at least at different extents. This effect resembles hormesis situations, where the cells’ machinery is triggered through the activation of different alert mechanisms, such as the modulation of stress resistance pathways, which may help the cells to cope with further damages.^[^ ^29^ ^]^ In such conditions, cells increase the production of several proteins including growth factors, phase II and antioxidant enzymes, and protein chaperones.^[^ ^29^ ^]^ Previous studies have been reporting several evidences of different phytochemicals and their neurohormetic effects that ultimately may increase the resistance to diseases (as reviewed in Mattson^[^ ^30^ ^]^). Moreover, hormetic phenomenon was also described in previous studies using simpler and more physiological metabolites like urolithin A that induced the transcription of mitochondrial biogenesis‐related genes,^[^ ^31^ ^]^ and protocatechuic, gallic, and vanillic acid by activating the sirtuin pathway.^[^ ^32^ ^]^ Definitely, phytochemicals at subtoxic doses may be able to activate adaptative cellular stress‐response pathways, driving the cells to protective effects. Our study gathers new clues about preconditioning evidence by physiological relevant LMWPM.

To test our hypothesis, a PCR array targeting major signal transduction pathways was designed and cat‐sulf and pyr‐sulf effects assessed in terms of gene expression. Interestingly, and despite the limitation of the biased choice of targets within the PCR array, gene ontology analysis revealed the basis of the biological function of the differentially expressed genes induced by both LMWPM: genes appeared to be involved in apoptosis regulation, cell homeostasis, and cell cycle process, suggesting that both LMWPM could impact cell machinery related to stress response. Pathway analysis identified some specific pathways that can be altered by the LMWPM. Cat‐sulf showed to be more effective in neuroprotection‐induced changes in genes regulating, for instance, neurotrophin signaling pathway, which plays an essential role in the proliferation, differentiation, and survival of neurons.^[^ ^33^ ^]^ Neurotrophins are also linked to MAPK and PI3K‐Akt signaling, supporting the hypothesis that such pathways can integrate signals from a range of stimuli and guide the cell to the appropriate response.^[^ ^34^, ^35^ ^]^ Moreover, autophagy regulation and protein processing also raised from the cellular pathway analysis for cat‐sulf and are in line with the normal cellular response to stress, whereas the same was not observed in pyr‐sulf treatment. These interconnected pathways may be explained by the PPI, not only those coded by the genes differentially expressed but also those that could interact with them, whose activity could be indirectly affected by cat‐sulf, impacting neuronal cell functions as well. From this analysis, AKT1, a critical mediator of growth factor‐induced neuronal survival, seems to have a central role, interacting with a huge number of other proteins. Its downregulation may affect several proteins and mechanisms such as, decreasing the phosphorylation of components of the apoptotic machinery, which in normal conditions would inactivate it.^[^ ^36^ ^]^ Also, ATF4, a TF associated to most of the pathways for which a higher number of genes were identified, is linked to cellular stress response. In turn, MAP3K1 protein, does not present many protein associations with differently expressed genes by cat‐sulf, but it is connected to MAPK signaling pathway together with central proteins like AKT1, ATF4, DDIT3, and MYC, suggesting its involvement in common cellular processes. Indeed, MAP3K1 works as an integration point for a variety of biochemical signals, driving the cell response either by activating ERK and NF‐κB pathways for promoting cell survival,^[^ ^37^ ^]^ or c‐Jun that may induce both pro‐ and anti‐apoptotic effects,^[^ ^38^ ^]^ depending on the stimuli. By docking analysis, we found a favorable binding capacity between JUN and cat‐sulf, which could elucidate the gene modulation induced by this metabolite. To the best of our knowledge, we are reporting for the first time a potential direct interaction of cat‐sulf with JUN, opening doors for new targeted studies. Also, MYC, one of the proteins associated to cell cycle, seems to be involved in central signaling pathways like MAPK and PI3K‐Akt and it is described as increased in degenerating neurons, supporting the hypothesis that neurodegeneration is related with an inappropriate cell cycle control, similarly to cellular proliferation in cancer.^[^ ^39^ ^]^ TP53, common to both LMWPM, has already been described as involved in protective mechanisms associated to (poly)phenols,^[^ ^40^, ^41^ ^]^ but it is not clear how this can affect downstream metabolism.

Like TFs, miRNAs may also interfere with gene expression by inducing their post‐transcriptional regulation, playing a key role in fundamental biological functions, including cells’ response to stress. Among the miRNA identified with higher number of target interactions with significantly altered genes, miR‐34a‐5p, let‐7c‐5p, miR‐212‐3p, and miR‐449a are described as being deregulated by an oxidative stress insult of MPP^+^.^[^ ^42^ ^]^ This deregulation in miRNA expression dynamics in response to oxidative stimulus may be associated with impairment of cell viability.^[^ ^42^ ^]^ For instance, the top predicted miRNA to be involved in the modulation triggered by both LMWPM, that belongs to the miR‐34a family, has been established as closely related to apoptosis induction via p53,^[^ ^43^, ^44^, ^45^ ^]^ and as suppressor of pro‐survival protein Bcl‐2 in the brain.^[^ ^46^ ^]^ Interestingly, miR‐34a appears to act mostly in the neurotoxic pathways triggering the same targets as the neurotoxic insult MPP^+^,^[^ ^47^ ^]^ supporting the idea of a preconditioning effect by LMWPM. In addition, miR‐876‐5p is involved in cell response to oxidative stress,^[^ ^42^ ^]^ and let‐7g family deregulation has been positively correlated to the prefrontal cortex and blood leukocytes of PD patients.^[^ ^48^ ^]^

Our analysis uncovered potential mechanisms underlying the distinct neuroprotective effects observed for each of the LMWPM. Focusing on cat‐sulf, it was clear the modulation of a number of genes working as ER stress sensors (like *ATF4*, *BECN1*, *ATG5*, *ATG12*, *DDIT3*, *AKT1*, *BCL2L1*, MAP3K1) that in fact might help the cells triggering the necessary mechanisms of survival to resist a stronger oxidative insult. When neurospheroids were exposed to MPP^+^ after LMWPM pretreatment, genes involved in apoptosis response, like *BCL2*, and mRNA expression of stress response‐related genes, like *ATF4*, *NQO1*, *GSR* and *HSP40*, emerged as significantly upregulated. Cat‐sulf significantly increased mRNA levels of the anti‐apoptotic *BCL2*, also increasing the ratio *BCL2*/*BAX* mRNA levels. In fact, modulation of apoptosis could be a mechanism of action by which cat‐sulf can be neuroprotective in a PD‐like scenario. Several other studies have highlighted the ability of distinct (poly)phenols, such as resveratrol, curcumin, or caffeic acid to mitigate neuronal damages through the upregulation of the Nrf2 pathway.^[^ ^49^, ^50^, ^51^ ^]^ Although some reports have demonstrated the neuroprotective effects of complex (poly)phenols, since they are not present at physiological concentrations in circulation, their relevance fades in opposition to data that reflect effects of physiological metabolites, as the LMWPM. A recent study has shown the potential of pyrogallol exerting anti‐inflammatory effects through the activation of Nrf2/HO‐1 pathway and inhibiting NF‐κB pathway.^[^ ^52^ ^]^ Yet, consistent data focusing on LMWPM is still lacking. Our results try to push forward the adoption of studies conditions as physiological as possible for the study of LMWPM, from the 3D cell model of PD to circulating (poly)phenol metabolites with biological relevance, at relevant concentrations.

Oxidative stress is a major factor contributing to the vulnerability of dopaminergic cells and beneficial effects of the induction of oxidative stress‐related genes towards PD are already described.^[^ ^53^ ^]^ The induction of *NQO1* gene expression of was shown to confer protection to dopaminergic neurons in both cell culture and animal models of PD.^[^ ^54^ ^]^ The upregulation of *NQO1* after 6 h of MPP^+^ lesion induced by the preincubation with circulating LMWPM, corroborates such observations.

Additionally, a decrease in GSH levels is one of the earliest biochemical alterations detected in association with PD and GSH itself regulates dopaminergic cell death through a wide variety of homeostatic processes.^[^ ^55^ ^]^ In fact, depletion of GSH was demonstrated to induce degeneration of nigral dopaminergic neurons in adult rats,^[^ ^56^ ^]^ possibly by promoting the increase of nitrosative stress.^[^ ^57^ ^]^ By stimulating the upregulation of the transcripts of glutathione reductase (*GSR*), the enzyme which converts GSSG into GSH, the LMWPM in study can be counteracting another important PD hallmark. Accordingly, both metabolites have shown a slight tendency to decrease the GSSG/GSH ratio that is increased by MPP^+^. Moreover, LMWPM appear to be also promoting cell's ability for protein glutathionylation, an important mechanism of post‐translational modification that protect cells from oxidative stress^[^ ^58^ ^]^ and tightly associated to dopaminergic neuron survival.^[^ ^59^ ^]^ Previous studies, using dietary (poly)phenols have also demonstrated the ability of quercetin, kaempferol, and apigenin flavonoids to increase intracellular GSH levels by promoting the transcription of g‐glutamylcysteine synthetase.^[^ ^60^ ^]^ Moreover, myricetin pretreatment prevented H_2_O_2_‐induced glutathione oxidation, decreasing also the ratio GSSG/GSH.^[^ ^61^ ^]^

Of relevance to the present work, the redox status of cells, namely in both cytosol and ER. In neurodegenerative diseases like PD, ER stress has been shown to deregulate redox homeostasis, promoting a dramatic increase in protein deposition due to increase of misfolded proteins aggregation.^[^ ^62^ ^]^ In fact, glutathione redox homeostasis seems to be essential to proteostasis maintenance through autophagy regulation.^[^ ^62^ ^]^ mRNA levels of *BECN1* did not change consistently with metabolite pretreatment and MPP^+^ and we observed a beneficial effect from both cat‐sulf and pyr‐sulf via upregulation of Hsp40 expression. Hsp40 is a co‐chaperone able to stimulate ATPase activity of Hsp70 (DnaK), which is protective in models of PD.^[^ ^63^, ^64^, ^65^ ^]^ Our previous data already reported the upregulation of Hsp40 in SK‐N‐MC cells after incubation with a blackberry‐digested polyphenol extract.^[^ ^66^ ^]^ Since these proteins often work in concert, the upregulation of Hsp40 transcripts can be suggestive of an Hsp40‐mediated activation of the regulatory mechanism of Hsp70, with ultimate neuroprotective effects.^[^ ^67^ ^]^

**Figure 11:**
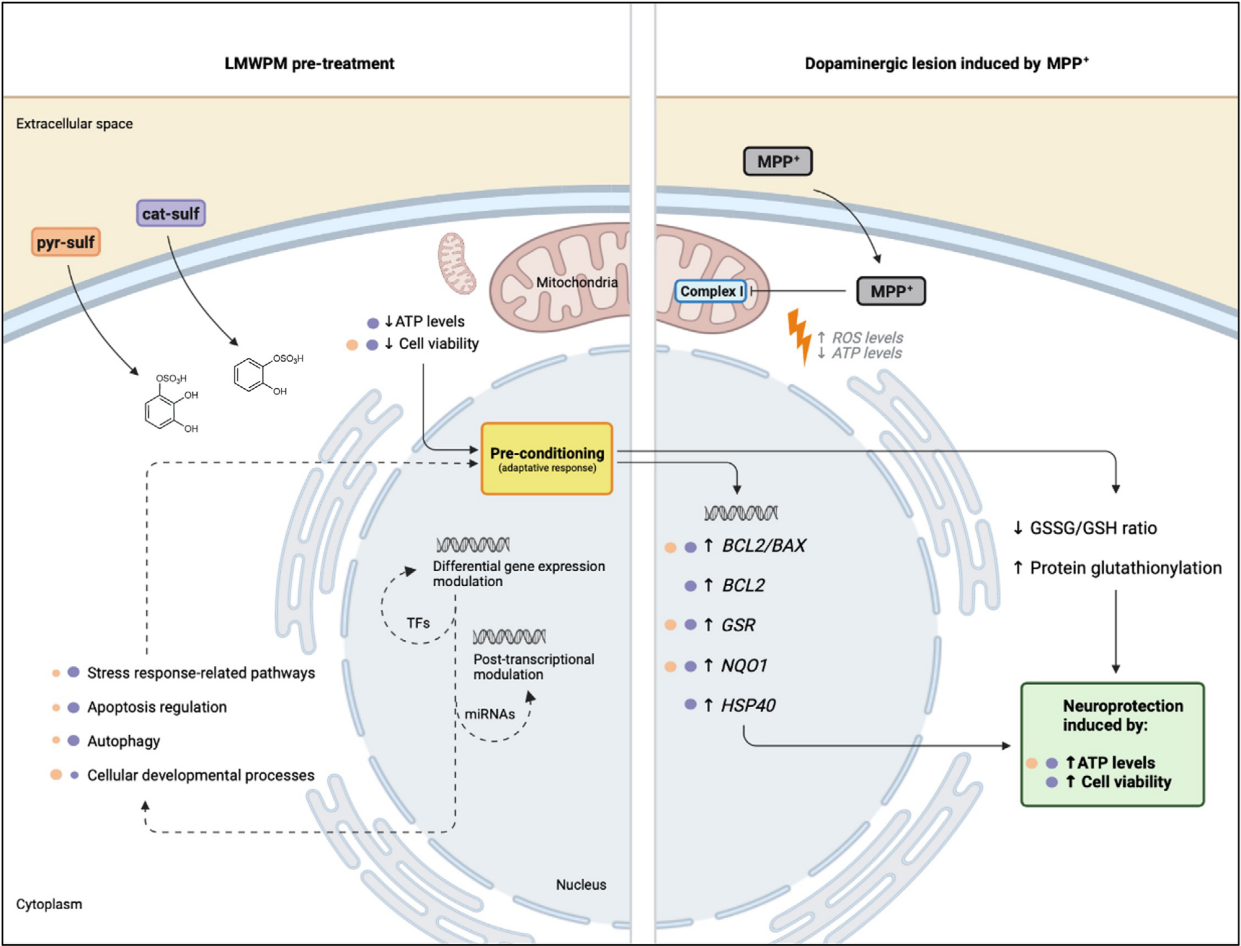
Graphical summary of LMWPM effects. Neuroprotective effects of both cat‐sulf and pyr‐sulf pretreatment in LUHMES neurospheroids challenged by MPP^+^. LMWPM induces differential modulation of gene expression and predictable differences in TFs, miRNAs regulation reflecting in different cellular pathways that overall promotes an adaptative response to better cope with MPP^+^ insult. Particularly, cat‐sulf, by positively modulating glutathione metabolism, apoptotic proteins balance and heat‐shock response, it promotes the increase of cell viability and ATP levels restoration. Purple (for cat‐sulf) and orange (for pyr‐sulf) circles represent the degree of involvement on such result; dashed arrows associate to multi‐omics analysis results; black arrows indicate biological data. Adapted from BioRender.com.

Our findings have pointed to the effects of LMWPM found in circulation, after a (poly)phenol‐rich supplementation, in triggering preconditioning mechanisms, which help dopaminergic neurons to cope with a later insult that induce oxidative stress, like MPP^+^ (**Figure** **11**). Considering changes in gene expression induced by cat‐sulf, we may argue that by stimulating ER stress sensors, this metabolite is able to modulate the cell machinery to better cope with a later oxidant exposure that may recruit a network of proteins, TFs and miRNAs. These targets that emerged from the bioinformatic analysis as altered by the LMWPM should be further explored in the context of dopaminergic lesion by MPP^+^. Nevertheless, the ability of such metabolites to tackle different cellular mechanisms involved in several chronic pathologies like neurodegenerative diseases, reinforce their potential as putative nutritional pleiotropic actors. Although it may be only a first glimpse towards the understanding of the molecular mechanisms underlying the benefits of the food we eat concerning age‐related diseases, our study can open the doors for additional studies taking advantage of these LMWPM, physiological concentrations, and disease‐relevant cell models.

## 4 Experimental Section

### Cell Culture and 2D Differentiation

Undifferentiated LUHMES cells were routinely cultivated in proliferation medium (Advanced DMEM/F12 (Gibco, Life Technologies) with 2 mM l‐Glutamine (Sigma‐Aldrich), 1xN2 Supplement (Gibco), and 0.04 µg mL^–1^ recombinant basic fibroblast growth factor (bFGF, R&D Systems)). For propagation flasks (Nunc) were precoated with 50 µg mL^–1^ poly‐l‐ornithine (PLO, Sigma‐Aldrich) and 1 µg mL^–1^ fibronectin (Sigma‐Aldrich).

### 3D Stirred Suspension Culture

Undifferentiated LUHMES cells were inoculated as a single cell suspension spinner vessel (Corning) equipped with a magnetic paddle impeller, at a density of 1.2 × 10^5^ cell mL^–1^ in 125 mL of proliferation medium and cultured in a humidified atmosphere of 5% CO_2_, at 37 °C. After 48 h of aggregation, 70% of medium was changed to differentiation medium (proliferation medium without bFGF and with 1 mM dibutyryl cAMP (Sigma‐Aldrich), 2 µg mL^–1^ tetracycline (Sigma‐Aldrich), and 2 ng mL^–1^ recombinant human glial cell line‐derived neurotrophic factor (GDNF, R&D Systems) to induce neuronal differentiation. Replacement of 50% of culture medium by fresh differentiation medium was performed every 2–3 days during a maximum of 14 days. Agitation rate was progressively increased from 60 to 70 rpm at differentiation step initiation, and up to 80 rpm by the end of the culture.

### Dosage Information

Differentiated neurospheroids (7 days of differentiation) were seeded in PLO‐fibronectin coated 96‐ (15 neurospheroid per well) or 12‐well plates (100 neurospheroid per well, Falcon). After 24 h, 200 nM idebenone (ide, provided by Grupo Tecnimede) or 5 µM of each LMWPM (cat‐sulf or pyr‐sulf) was added and, after 24 h of incubation, medium was removed and new differentiation medium containing 5 µM of MPP^+^ (Sigma‐Aldrich) was added for different timepoints. Cell viability and ATP levels were assessed 24 h after injury, while remaining parameters were assessed after 0, 2, 6, and 24 h of injury with MPP^+^.

### Cell Viability Assays

Neurospheroids were distributed in precoated 96‐well plates in differentiation medium. At endpoint, cell viability was assessed by Presto Blue assay (Invitrogen), and intracellular ATP levels by CellTiter Glo (Promega) according to manufacturer's instructions. Values were calculated as a percentage relatively to control cells (untreated neurospheroids).

### Gene Expression

Neurospheroids were distributed in precoated 12‐well plates. Total RNA was extracted from neurospheroids with High Pure RNA Isolation kit (Roche) and quantified using NanoRop 2000c (ThermoScientific). Reverse transcription was performed with Transcriptor High Fidelity cDNA Synthesis kit (Roche), and qPCR analysis was performed as described.^[^ ^68^ ^]^ List of primers used and sequences is presented in Table S1, Supporting Information. RealTime ready assays from Universal Probe Library (Roche) were used with forward and reverse primers (400 nM) and fluorescently labeled hydrolysis probes (200 nM) lyophilized in a Custom Panel 384 (configuration no. 100127094, Roche, Table S2, Supporting Information), performed according to manufacturer's instructions. Results were processed using the 2^–∆∆CT^ method for relative gene expression analysis. Changes in gene expression were normalized using the housekeeping genes *RPL22*, in the case of RT‐qPCR, and *GAPDH*, *B2M*, and *ACTB*, in the case of RealTime Ready assay.

### Transcriptomics Analyses of the Genomic Modifications

Gene ontology enrichment analysis was performed for the identified differentially expressed genes using ShinyGO (http://bioinformatics.sdstate.edu/go/).^[^ ^69^ ^]^ Cellular pathways were explored using GeneTRial2 software (https://genetrail2.bioinf.uni‐sb.de).^[^ ^70^ ^]^ Datasets were analyzed for KEGG and BioCarta pathway databases. Network of pathways, their interactions, and genes involved with each pathway were also searched using ClueGO Cytoscape (v 3.7.2) to create and visualize functionally grouped networks.^[^ ^71^ ^]^ Protein–protein interactions (PPI) between the proteins that were coded by the differentially expressed genes, including their neighboring proteins, was conducted using STRING (v10.5, (https://string‐db.org/),^[^ ^72^ ^]^ considering: confidence—text‐mining, experiments, databases, coexpression; high confidence—0.700; no more than 10 interactions in the first shell and no interactions in the second shell, without clustering. Genes identified in this PPI network were applied to pathway analysis using KEGG mapping analysis tool.^[^ ^73^ ^]^

Transcription factors (TFs) potentially involved in the transcriptional regulation of the identified genes were identified using the TTRUST database (https://www.grnpedia.org/trrust/).^[^ ^74^ ^]^ Potential interactions of the metabolites with TFs were assessed using SwissDock (http://www.swissdock.ch/docking) molecular modeling tool. The 3D structures of the proteins were obtained from Uniprot database and the chemical structures of the metabolites were obtained from PubChem database.

Search for miRNAs potentially involved in post‐transcriptional regulation of the differentially expressed genes was performed with MIcroRNA ENrichiment TURned NETwork (MIENTURNET), used for miRNA‐target enrichment and network‐based analysis (http://userver.bio.uniroma1.it/apps/mienturnet/).^[^ ^75^ ^]^ Intersections between differentially expressed genes with those identified as involved in neurodegenerative diseases and PD have been searched using Comparative Toxicogenomics Database (http://ctdbase.org).^[^ ^76^ ^]^

### Determination of Thiolomic Profile

The cysteine‐related thiolomic profile was obtained through the quantification of cysteine (Cys) and glutathione (GSH) fractions: total and free total, as previously reported,^[^ ^77^ ^]^ but adapted for cell culture. Briefly, neurospheroids pellets were homogenized with 100 µL of 0.01% Triton X‐100 in PBS and centrifuged (2 min, 10 600 × *g*, at 4 °C). Thiols were quantified by HPLC with fluorescence detection (HPLC‐FD, Shimadzu Scientific Instruments Inc.), after sample pretreatment for the separation of the different pools.^[^ ^78^ ^]^ The total fraction of each thiol represented the sum of free reduced (RSH) + free oxidized (RSSR) + protein bound pools (RSSP). The free total fraction comprised the RSH + RSSR fractions from each thiol. The protein‐bound RSSP fraction was obtained by subtracting the total free fraction to the total fraction.

### Glutathione (GSH) and Glutathione Disulfide (GSSG) Quantification

GSH and GSSG were quantified by HPLC after derivatization with ortho‐phthalaldehyde as previously described.^[^ ^79^ ^]^

### Statistical Analysis

Statistical analysis was performed using GraphPad Prism 9 software. Differences in gene expression from PCR array were assessed by Student's *t*‐test. RT‐qPCR, ATP fold change, neurotoxicity, and neuroprotection data are mean ± SEM from at least three independent experiments (independent spinner vessels) performed with technical replicates. One‐way or two‐way ANOVA analysis with Tukey's and Bonferroni's post multiple comparison test, respectively, were performed to assess statistical differences between conditions.

## Conflict of Interest

The authors declare no conflict of interest.

## Authors Contributions

R.C., I.F., and C.N.S. designed the study and drafted the manuscript; R.C., I.F., A.P.T., J.G.‐P., C.O.S., and S.A.P. conducted cell experiments; R.C., I.F., C.O.S., S.A.P., and D.M. analyzed the results and interpreted the data; S.A.P. D.M., M.L., C.B., and C.N.S. revised the manuscript. All authors read and approved the final manuscript.

## Supporting information

Supporting Information

## Data Availability

The data that support the findings of this study are available from the corresponding author upon reasonable request.
