# Supplementary material for: Circulating (Poly)phenol Metabolites: Neuroprotection in a 3D Cell Model of Parkinson's Disease
Source: Mol Nutr Food Res. 2022 Jan 8;66(21):2100959. doi: 10.1002/mnfr.202100959 (PMC9788306; doi:10.1002/mnfr.202100959)
Supplement: Supplementary file 1 — Supporting Information [file MNFR-66-2100959-s001.pdf]

Supporting Information

Circulating (poly)phenol metabolites: neuroprotection in a 3D cell model of Parkinson's disease

1. Supplementary data

1.1. Neurospheroids monitoring along time

At different time points along culture progression, viability of differentiated neurospheroids was assessed using a fluorescence microscopy-based method, the fluoresceine diacetate (FDA)/propidium iodide (PI) assay.[1] Moreover, cell proliferation was also assessed by fluorescence microscopy using Click-iT Plus EdU AlexaFluor™ 488 Assay kit (Life Technologies). Neurospheroids sampling was performed along differentiation and neurospheroids disrupted in 0.1 M citric acid with 1% Triton X-100, at 37 °C, overnight, and nuclei stained with 0.1 % crystal violet and counted in a Fuchs–Rosenthal haemocytometer chamber.[2] A sample size of at least 1 mL of culture was used to reliably determine cell concentration, neurospheroids counting and perform aggregate size measurements using ImageJ software.

1.2. Neurospheroids synaptic activity

Evoked synaptic activity of Neurospheroids was assessed by the method of Gaffield 2006,[3] and already reported by us.[4] LUHMES Neurospheroids were collected at 0 and 7 days of differentiation and seeded in PLO-fibronectin pre-coated glass coverslips, and incubated with 100 mM KCl buffer (5 mM HEPES-NaOH, pH 7.4; 10 mM glucose; 2.5 mM calcium chloride; 1 mM magnesium chloride; 100 mM potassium chloride; 37 mM sodium chloride) for 5 min. Afterwards, 100 mM KCl buffer was removed and Neurospheroids incubated with 10 µM FM-1-43 dye (Invitrogen) in 5 mM KCl buffer (5 mM HEPES-NaOH, pH 7.4; 10 mM glucose; 2.5 mM calcium chloride; 1 mM magnesium chloride; 5 mM potassium chloride; 37 mM sodium chloride) for 15 min. Neurospheroids were washed for 1 min with 5 mM KCl buffer with ADAVASEP-7 (Sigma) followed by three washes with 5 mM KCl buffer. Exocytosis was stimulated with 100 mM KCl buffer and samples were visualized live using a fluorescence microscope (Leica DMI6000) in order to monitor the decrease in fluorescence intensity over time. Fluorescence intensity was measured using ImageJ software.

Spontaneous synaptic activity of LUHMES neurospheroids was assessed by their response in calcium assay.[5] Neurospheroids with 7 days of differentiation were incubated with 1x Fluo-4 Direct calcium reagent (Invitrogen) for 30 min at 37 °C, 5% CO_2 and for 15 min at RT. Samples were then imaged live using spinning disk microscopy (Nikon Eclipse Ti-E, confocal scanner: Yokogawa CSU-x1). Fluorescence change over time is defined as $\Delta F/F0 = (F-F0)/F0$, where F is the fluorescence at any time point, and F0 the baseline fluorescence determined by baseline fitting across the whole movie for each cell using SparkMaster plugin of ImageJ software.

1.3. Immunofluorescence microscopy

Neurospheroids were collected at 7 days of differentiation and processed for immunofluorescence staining.[4] Briefly, neurospheroids were fixed in 4% (w/v) paraformaldehyde (Sigma-Aldrich) solution in PBS with 4% (w/v) sucrose and processed directly for immunostaining. The primary antibodies used for cell characterization were anti-βIII-tubulin (βIII-tub, Millipore), anti-glial fibrillary acidic protein (GFAP, DAKO) and anti-tyrosine hydroxylase (TH, Santa Cruz Biotechnology). The secondary antibodies used were goat anti-mouse IgG-AlexaFluor 488 and goat anti-rabbit IgG AlexaFluor 594 (Life Technologies). Cell nuclei were counterstained with 4',6-diamidino-2-phenylindole (DAPI, Life Technologies). Preparations were visualized using a point-scan confocal (Leica SP5) microscope. Merge between channels, maximum z-projections, and orthogonal projections, as well as linear brightness and contrast adjustment of the images were created using ImageJ software.

1.4. Western blot

Neurospheroids were lysed in TX-100 lysis buffer (50 mM Tris, 5 mM EDTA, 150 mM NaCl, 1% Triton X-100 and 1x complete protease inhibitors cocktail (Roche)) and protein quantification was carried out using the Micro BCA™ Protein Assay kit (Thermo Fisher). Protein samples were subjected to a gel electrophoresis using a NuPAGE 4-12% Bis-Tris Gel with MES running buffer (Invitrogen) and electrophoretically transferred to polyvinylidene difluoride (PVDF) membrane. Membranes were blocked with 5% non-fat dried milk powder in 0.1% Tween-20 in PBS (blocking solution) for 1h, followed by incubation with primary antibodies overnight at 4 °C, diluted in blocking solution and then with secondary antibodies (horseradish peroxidase-conjugated, ECL anti-mouse IgG or anti-rabbit

IgG; GE Healthcare) diluted 1:5000 in blocking solution, for 2h at RT. Anti-α-tubulin or anti-GAPDH antibodies were used as control to confirm equal loading of total protein. Membranes were developed using Amersham ECL Prime Western Blotting Detection Reagent (GE Healthcare) and visualized using a ChemiDocTM XRS+ System (BioRad). Primary antibodies used for protein detection were anti-βIII-tubulin (Millipore), anti-synaptophysisn (Syn, Millipore), anti-GFAP (DAKO), anti-TH (Santa Cruz), anti-Nestin (Millipore), anti-glutamate transporter (GLT-1, Millipore) and anti- Pituitary homeobox-3 (Pitx3, Abcam).

Supplementary material

Table S1. List of primers used for RT-qPCR analysis.

Gene	Protein	Primers forward (top) and reverse (bottom)
RPL22	Ribosomal protein L22 (housekeeping gene)	CACGAAGGAGGAGTGACTGG<br>TGTGGCACACCACTGACATT
TUJ1	βIII-tubulin	GGGCCTTTGGACATCTCTTC<br>CCTCCGTGTAGTGACCCTTG
TH	Tyrosine hydroxylase	AGCCCTACCAAGACCAGACG<br>GCGTGTACGGGTCGAACTT
DAT	Dopamine transporter	ACCTTCCTCCTGTCCCTGTT<br>CACCATAGAACCAGGCCACT
SYP	Synaptophysin	TTTGTGAAGGTGCTGCAATG<br>GCTGAGGTCACTCTCGGTCT
NES	Nestin	TAAGGTGAAAAGGGGTGTGG<br>GCAAGAGATTCCCTTTGCAG
GFAP	Glial fibrillary acid protein	AGAGAGGTCAAGCCCAGGAG<br>GGTCACCCACAACCCCTACT
ATF4	Activating transcription factor 4	GGCTGGCTGTGGATGGGTTG<br>CTCCTGGACTAGGGGGGCAA
GSR	Glutathione reductase	CGTGGAGGTGCTGAAGTTCTC<br>TCATGGTCATGACTGGTAGCC
NQO1	NAD(P)H quinone dehydrogenase 1	TGACATATAGCATTGGGCACAC<br>TTCTCCTCATCCTGTACCTCTT
HSP40	DnaJ (heat shock protein 40 kD)	AGGAAGGCCTAAAGGGGAGT<br>AGGGATTTCTGCCACCGAAG
BCL-2	B-cell lymphoma 2	ATCGCCCTGTGGATGACTGAG<br>CAGCCAGGAGAAATCAAACAGAGG
BAX	BCL-2 associated X protein	TGGAGCTGCAGAGGATGATTG<br>GAAGTTGCCGTCAGAAAACATG
BECN1	Beclin-1	CGTGGAAAAGAACCGCAAGAT<br>GCTGCGTCTGGGCATAACG
MAP2K1	Mitogen-Activated Protein Kinase Kinase 1	AACTCTCCGTACATCGTGGG<br>TGATCTTGTGCTTCTCCCTCAG

Table S2. RealTime Ready Custom panel information. Assay ID, gene symbol (*H. sapiens*), alias and description according to https://configurator.realtimeready.roche.com/.

Assay ID	Gene Symbol	Alias	Description
138446	ACSL3	ACS3; FACL3; PRO2194	acyl-CoA synthetase long-chain family member 3 [Source:HGNC Symbol;Acc:3570]
127578	ACSL4	ACS4; FACL4; LACS4; MRX63; MRX68	acyl-CoA synthetase long-chain family member 4 [Source:HGNC Symbol;Acc:3571]
119501	ACSL5	ACS2; ACS5; FACL5	acyl-CoA synthetase long-chain family member 5 [Source:HGNC Symbol;Acc:16526]
100816	AKT1	AKT; MGC99656; PKB; PKB-ALPHA; PRKBA; RAC; RAC-ALPHA	v-akt murine thymoma viral oncogene homolog 1 [Source:HGNC Symbol;Acc:391]
114661	ATF4	CREB-2; CREB2; TAXREB67; TXREB	activating transcription factor 4 (tax-responsive enhancer element B67) [Source:HGNC Symbol;Acc:786]
115129	ATF6	ATF6A	activating transcription factor 6 [Source:HGNC Symbol;Acc:791]
118103	ATG12	APG12; APG12L; FBR93; HAPG12	ATG12 autophagy related 12 homolog (S. cerevisiae) [Source:HGNC Symbol;Acc:588]
125999	ATG5	APG5; APG5-LIKE; APG5L; ASP; hAPG5	ATG5 autophagy related 5 homolog (S. cerevisiae) [Source:HGNC Symbol;Acc:589]
120541	ATG7	APG7-LIKE; APG7L; DKFZp434N0735; GSA7	ATG7 autophagy related 7 homolog (S. cerevisiae) [Source:HGNC Symbol;Acc:16935]
102998	AXIN2	AXIL; DKFZp781B0869; MGC10366; MGC126582	axin 2 [Source:HGNC Symbol;Acc:904]
142318	BAX	BCL2L4	BCL2-associated X protein [Source:HGNC Symbol;Acc:959]
142759	BBC3	FLJ42994; JFY1; PUMA	BCL2 binding component 3 [Source:HGNC Symbol;Acc:17868]
100083	BCL2	Bcl-2	B-cell CLL/lymphoma 2 [Source:HGNC Symbol;Acc:990]
100085	BCL2A1	ACC-1; ACC-2; BCL2L5; BFL1; GRS; HBPA1	BCL2-related protein A1 [Source:HGNC Symbol;Acc:991]
100088	BCL2L1	Bcl-X; bcl-xL; BCL-XL/S; bcl-xS; BCL2L; BCLX; BCLXL; BCLXS; DKFZp781P2092	BCL2-like 1 [Source:HGNC Symbol;Acc:992]
100115	BECN1	ATG6; beclin1; VPS30	beclin 1, autophagy related [Source:HGNC Symbol;Acc:1034]
100122	BID	FP497; MGC15319; MGC42355	BH3 interacting domain death agonist [Source:HGNC Symbol;Acc:1050]
100135	BIRC3	AIP1; API2; c-IAP2; cIAP2; HAIP1; hiap-1; HIAP1; MALT2; MIHC; RNF49	baculoviral IAP repeat-containing 3 [Source:HGNC Symbol;Acc:591]
146274	BMP2	BMP2A	bone morphogenetic protein 2 [Source:HGNC Symbol;Acc:1069]
146294	BMP4	BMP2B; BMP2B1; MCOPS6; OFC11; ZYME	bone morphogenetic protein 4 [Source:HGNC Symbol;Acc:1071]
110980	BTG2	MGC126063; MGC126064; PC3; TIS21	BTG family, member 2 [Source:HGNC Symbol;Acc:1131]
-	CALR	cC1qR; CRT; FLJ26680; RO; SSA	calreticulin [Source:HGNC Symbol;Acc:1455]

113395	CCL5	D17S136E; MGC17164; RANTES; SCYA5; SISd; TCP228	chemokine (C-C motif) ligand 5 [Source:HGNC Symbol;Acc:10632]
142502	CCND1	BCL1; D11S287E; PRAD1; U21B31	cyclin D1 [Source:HGNC Symbol;Acc:1582]
101384	CCND2	KIAK0002; MGC102758	cyclin D2 [Source:HGNC Symbol;Acc:1583]
144097	CDKN1A	CAP20; CDKN1; CIP1; MDA-6; P21; p21CIP1; p21Cip1/Waf1; SDI1; WAF1	cyclin-dependent kinase inhibitor 1A (p21, Cip1) [Source:HGNC Symbol;Acc:1784]
100855	CDKN1B	CDKN4; KIP1; MEN1B; MEN4; P27KIP1	cyclin-dependent kinase inhibitor 1B (p27, Kip1) [Source:HGNC Symbol;Acc:1785]
-	CEBPD	C/EBP-delta; CELF; CRP3; NF-IL6-beta	CCAAT/enhancer binding protein (C/EBP), delta [Source:HGNC Symbol;Acc:1835]
113740	CPT2	CPT1; CPTASE	carnitine palmitoyltransferase 2 [Source:HGNC Symbol;Acc:2330]
141077	CSF1	M-CSF; MCSF; MGC31930	colony stimulating factor 1 (macrophage) [Source:HGNC Symbol;Acc:2432]
116795	DAB2	DOC-2; DOC2; FLJ26626	disabled homolog 2, mitogen-responsive phosphoprotein (Drosophila) [Source:HGNC Symbol;Acc:2662]
100355	DDIT3	CEBPZ; CHOP; CHOP-10; CHOP10; GADD153; MGC4154	DNA-damage-inducible transcript 3 [Source:HGNC Symbol;Acc:2726]
115809	DNAJC3	FLJ21288; HP58; P58; P58IPK; PRKRI	DnaJ (Hsp40) homolog, subfamily C, member 3 [Source:HGNC Symbol;Acc:9439]
143550	EGFR	ERBB; ERBB1; HER1; mENA; PIG61	epidermal growth factor receptor [Source:HGNC Symbol;Acc:3236]
148159	EMP1	CL-20; EMP-1; TMP	epithelial membrane protein 1 [Source:HGNC Symbol;Acc:3333]
103087	FABP1	FABPL; L-FABP	fatty acid binding protein 1, liver [Source:HGNC Symbol;Acc:3555]
145362	FAS	ALPS1A; APO-1; APT1; CD95; FAS1; FASTM; TNFRSF6	Fas (TNF receptor superfamily, member 6) [Source:HGNC Symbol;Acc:11920]
-	FCER2	CD23; CD23A; CLEC4J; FCE2; IGEBF	Fc fragment of IgE, low affinity II, receptor for (CD23) [Source:HGNC Symbol;Acc:3612]
104340	FOSL1	FRA; fra-1; FRA1	FOS-like antigen 1 [Source:HGNC Symbol;Acc:13718]
117090	FTH1	FHC; FTH; FTHL6; MGC104426; PIG15; PLIF	ferritin, heavy polypeptide 1 [Source:HGNC Symbol;Acc:3976]
101471	GADD45A	DDIT1; GADD45	growth arrest and DNA-damage-inducible, alpha [Source:HGNC Symbol;Acc:4095]
115714	GADD45B	DKFZP566B133; GADD45BETA; MYD118	growth arrest and DNA-damage-inducible, beta [Source:HGNC Symbol;Acc:4096]
110864	GATA3	HDR; MGC2346; MGC5199; MGC5445	GATA binding protein 3 [Source:HGNC Symbol;Acc:4172]
147654	GCLC	GCL; GCS; GLCL; GLCLC	glutamate-cysteine ligase, catalytic subunit [Source:HGNC Symbol;Acc:4311]
114136	GCLM	GLCLR	glutamate-cysteine ligase, modifier subunit [Source:HGNC Symbol;Acc:4312]
111427	GSR	MGC78522	glutathione reductase [Source:HGNC Symbol;Acc:4623]
102005	HERPUD1	HERP; KIAA0025; Mif1; SUP	homocysteine-inducible, endoplasmic reticulum stress-inducible, ubiquitin-like domain member 1 [Source:HGNC Symbol;Acc:13744]

142326	HES1	bHLHb39; FLJ20408; HES-1; HHL; HRY	hairy and enhancer of split 1, (Drosophila) [Source:HGNC Symbol;Acc:5192]
-	HES5	bHLHb38	hairy and enhancer of split 5 (Drosophila) [Source:HGNC Symbol;Acc:19764]
142325	HEY1	BHLHb31; CHF-2; CHF2; HERP2; HESR-1; HESR1; HRT-1; MGC1274; OAF1	hairy/enhancer-of-split related with YRPW motif 1 [Source:HGNC Symbol;Acc:4880]
112778	HEY2	bHLHb32; CHF1; GRIDLOCK; GRL; HERP1; HESR2; HRT2; MGC10720	hairy/enhancer-of-split related with YRPW motif 2 [Source:HGNC Symbol;Acc:4881]
137073	HEYL	bHLHb33; HEY3; HRT3; MGC12623	hairy/enhancer-of-split related with YRPW motif-like [Source:HGNC Symbol;Acc:4882]
110977	HMOX1	bK286B10; HO-1; HSP32	heme oxygenase (decycling) 1 [Source:HGNC Symbol;Acc:5013]
110865	HSP90AA1	FLJ31884; HSP86; Hsp89; HSP89A; Hsp90; HSP90A; HSP90AA2; HSP90N; HSPC1; HSPCA; HSPCAL1; HSPCAL4; HSPN; LAP2	heat shock protein 90kDa alpha (cytosolic), class A member 2 [Source:HGNC Symbol;Acc:5256]
100489	HSP90B1	ECGP; GP96; GRP94; TRA1	heat shock protein 90kDa beta (Grp94), member 1 [Source:HGNC Symbol;Acc:12028]
110730	HSPA4	APG-2; HS24/P52; hsp70; hsp70RY; MGC131852; RY	heat shock 70kDa protein 4 [Source:HGNC Symbol;Acc:5237]
110805	HSPA5	BiP; FLJ26106; GRP78; MIF2	heat shock 70kDa protein 5 (glucose-regulated protein, 78kDa) [Source:HGNC Symbol;Acc:5238]
100945	ICAM1	BB2; CD54; P3.58	intercellular adhesion molecule 1 [Source:HGNC Symbol;Acc:5344]
104631	ID1	bHLHb24; dJ857M17.1; ID	inhibitor of DNA binding 1, dominant negative helix-loop-helix protein [Source:HGNC Symbol;Acc:5360]
110609	IFNG	IFG; IFI	interferon, gamma [Source:HGNC Symbol;Acc:5438]
117683	IFRD1	PC4; TIS7	interferon-related developmental regulator 1 [Source:HGNC Symbol;Acc:5456]
144798	IRF1	IRF-1; MAR	interferon regulatory factor 1 [Source:HGNC Symbol;Acc:6116]
108043	JAG1	AGS; AHD; AWS; CD339; HJ1; JAGL1; MGC104644	jagged 1 [Source:HGNC Symbol;Acc:6188]
112383	LFNG	SCDO3	LFNG O-fucosylpeptide 3-beta-N-acetylglucosaminyltransferase [Source:HGNC Symbol;Acc:6560]
146268	LRG1	HMFT1766; LRG	leucine-rich alpha-2-glycoprotein 1 [Source:HGNC Symbol;Acc:29480]
106176	MAP3K1	MAPKKK1; MEKK; MEKK1	mitogen-activated protein kinase kinase kinase 1 [Source:HGNC Symbol;Acc:6848]
102930	MCL1	bcl2-L-3; BCL2L3; EAT; Mcl-1; MCL1-ES; mcl1/EAT; MCL1L; MCL1S; MGC104264; MGC1839; TM	myeloid cell leukemia sequence 1 (BCL2-related) [Source:HGNC Symbol;Acc:6943]
104396	MMP7	MMP-7; MPSL1; PUMP-1	matrix metallopeptidase 7 (matrilysin, uterine) [Source:HGNC Symbol;Acc:7174]
100977	MYC	bHLHe39; c-Myc; MRTL	v-myc myelocytomatosis viral oncogene homolog (avian) [Source:HGNC Symbol;Acc:7553]
142322	NOTCH1	hN1; TAN1	notch 1 [Source:HGNC Symbol;Acc:7881]

147227	NQO1	DHQU; DIA4; DTD; NMOR1; NMORI; QR1	NAD(P)H dehydrogenase, quinone 1 [Source:HGNC Symbol;Acc:2874]
113212	OLR1	CLEC8A; LOX-1; LOX1; LOXIN; SCARE1; SLOX1	oxidized low density lipoprotein (lectin-like) receptor 1 [Source:HGNC Symbol;Acc:8133]
101524	PCNA	MGC8367; PCNAAS	PCNA antisense RNA (non-protein coding) [Source:HGNC Symbol;Acc:37184]
104411	PPARD	FAAR; MGC3931; NR1C2; NUC1; NUCI; NUCII; PPARB	peroxisome proliferator-activated receptor delta [Source:HGNC Symbol;Acc:9235]
111252	PTCH1	BCNS; FLJ26746; FLJ42602; HPE7; NBCCS; PTC; PTC1; PTCH; PTCH11	patched 1 [Source:HGNC Symbol;Acc:9585]
105606	RAF1	c-Raf; CRAF; NS5; Raf-1	v-raf-1 murine leukemia viral oncogene homolog 1 [Source:HGNC Symbol;Acc:9829]
101596	RB1	OSRC; p105-Rb; pp110; pRb; RB	retinoblastoma 1 [Source:HGNC Symbol;Acc:9884]
117069	SLC27A4	ACSVL4; FATP4; IPS	solute carrier family 27 (fatty acid transporter), member 4 [Source:HGNC Symbol;Acc:10998]
101122	SOCS3	ATOD4; CIS3; Cish3; MGC71791; SOCS-3; SSI-3; SSI3	suppressor of cytokine signaling 3 [Source:HGNC Symbol;Acc:19391]
119555	SORBS1	CAP; DKFZp451C066; DKFZp586P1422; FLAF2; FLJ12406; KIAA1296; ponsin; R85FL; SH3D5; sh3p12; SORB1	sorbin and SH3 domain containing 1 [Source:HGNC Symbol;Acc:14565]
113560	SQSTM1	A170; OSIL; p60; p62; p62B; PDB3; ZIP3	sequestosome 1 [Source:HGNC Symbol;Acc:11280]
101180	STAT1	DKFZp686B04100; ISGF-3; STAT91	signal transducer and activator of transcription 1, 91kDa [Source:HGNC Symbol;Acc:11362]
147880	TNF	DIF; TNF-alpha; TNFA; TNFSF2	Tumor necrosis factor Precursor (TNF-alpha)(Tumor necrosis factor ligand superfamily member 2)(TNF-a)(Cachectin) [Contains Tumor necrosis factor, membrane form;Tumor necrosis factor, soluble form] [Source:UniProtKB/Swiss-Prot;Acc:P01375]
101266	TNFSF10	Apo-2L; APO2L; CD253; TL2; TRAIL	tumor necrosis factor (ligand) superfamily, member 10 [Source:HGNC Symbol;Acc:11925]
116591	TXNL4B	Dim2; DLP; FLJ20511	thioredoxin-like 4B [Source:HGNC Symbol;Acc:26041]
114835	TXNRD1	GRIM-12; MGC9145; TR; TR1; Trxr1; TXNR	thioredoxin reductase 1 [Source:HGNC Symbol;Acc:12437]
109914	ULK1	ATG1; ATG1A; FLJ38455; FLJ46475; Unc51	unc-51-like kinase 1 (C. elegans) [Source:HGNC Symbol;Acc:12558]
146965	WISP1	CCN4; WISP1c; WISP1i; WISP1tc	WNT1 inducible signaling pathway protein 1 [Source:HGNC Symbol;Acc:12769]
104468	WNT1	INT1	wingless-type MMTV integration site family, member 1 [Source:HGNC Symbol;Acc:12774]
104488	WNT2B	WNT13; XWNT2	wingless-type MMTV integration site family, member 2B [Source:HGNC Symbol;Acc:12781]
104494	WNT3A	MGC119418; MGC119419; MGC119420	wingless-type MMTV integration site family, member 3A [Source:HGNC Symbol;Acc:15983]
146266	WNT5A	hWNT5A	wingless-type MMTV integration site family, member 5A [Source:HGNC Symbol;Acc:12784]

47821	WNT6		wingless-type MMTV integration site family, member 6 [Source:HGNC Symbol;Acc:12785]
102065	B2M		beta-2-microglobulin [Source:HGNC Symbol;Acc:914]
143636	ACTB	PS1TP5BP1	actin, beta [Source:HGNC Symbol;Acc:132]
141139	GAPDH	G3PD; GAPD; MGC88685	glyceraldehyde-3-phosphate dehydrogenase [Source:HGNC Symbol;Acc:4141]

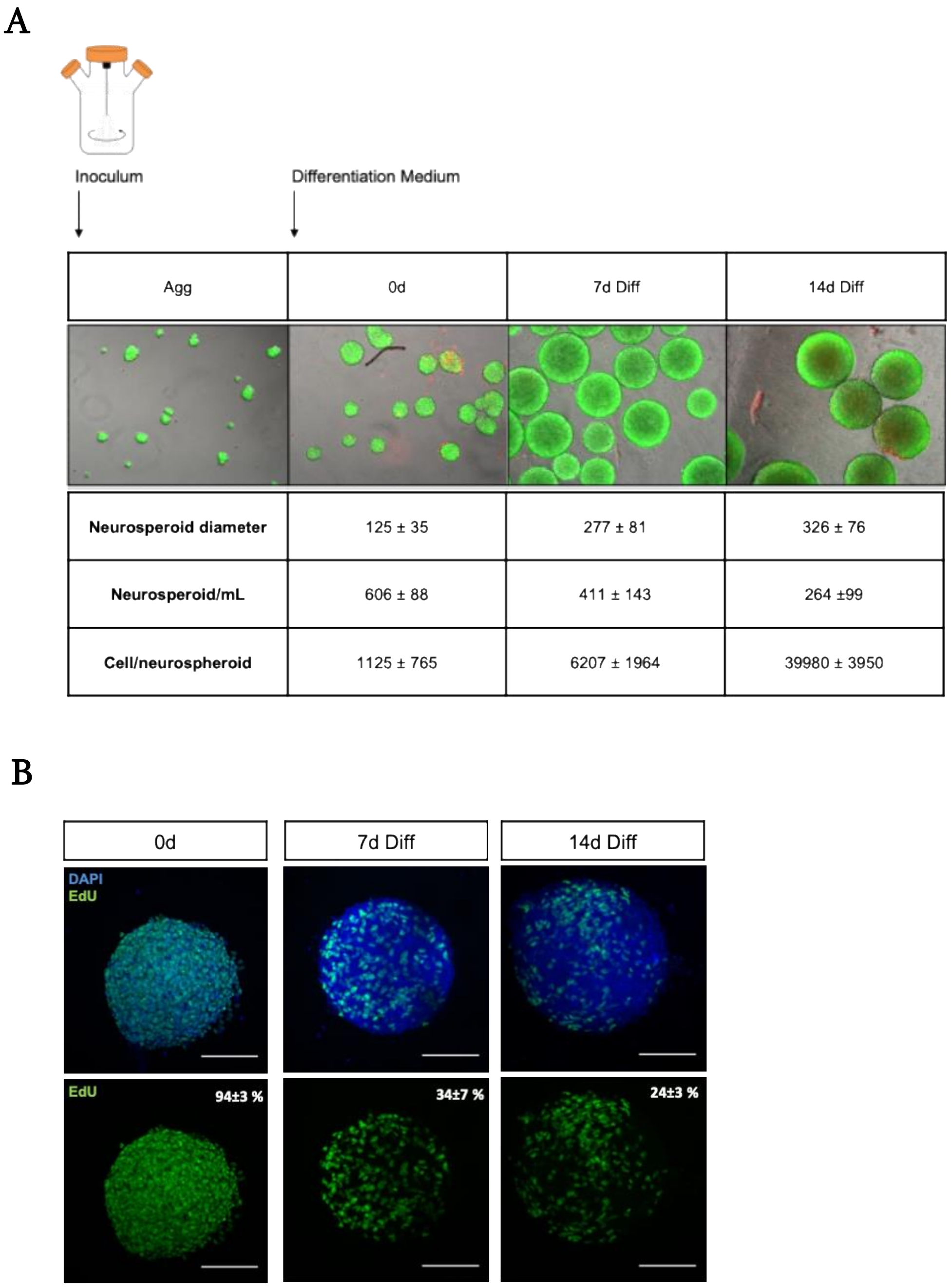

Figure S1. LUHMES neurospheroids cell culture characterization along time. Cells were inoculated in stirred suspension culture systems and aggregated (Agg) for 2 days. Dopaminergic differentiation was induced by removing bFGF from medium and by introduction of human GDNF, cAMP and tetracycline for 14 days (Differentiation Medium). (**A**) Representative images of the culture status at the indicated differentiation (Diff) days: fluorescent live/dead assay using fluorescein diacetate (FDA) for identification of live cells (green) and propidium iodide (PI) for identification of dead cells (red). Neurospheroids diameter (µm) profile and neurospheroids concentration along differentiation. (**B**) Representative images of neurospheroids proliferation status at the indicated differentiation (Diff) days and percentage of proliferative cells (EdU positive cells): EdU labeling of proliferative cells (green) and nuclei counterstain with DAPI (blue). Data are mean ± SD of three independent cultures. Scale bar: 100 µm.

A

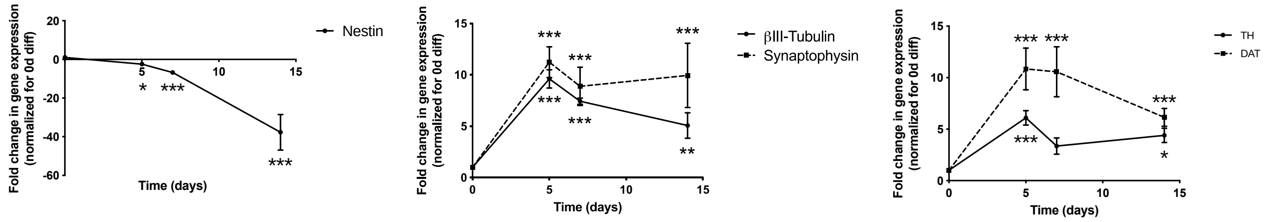

B

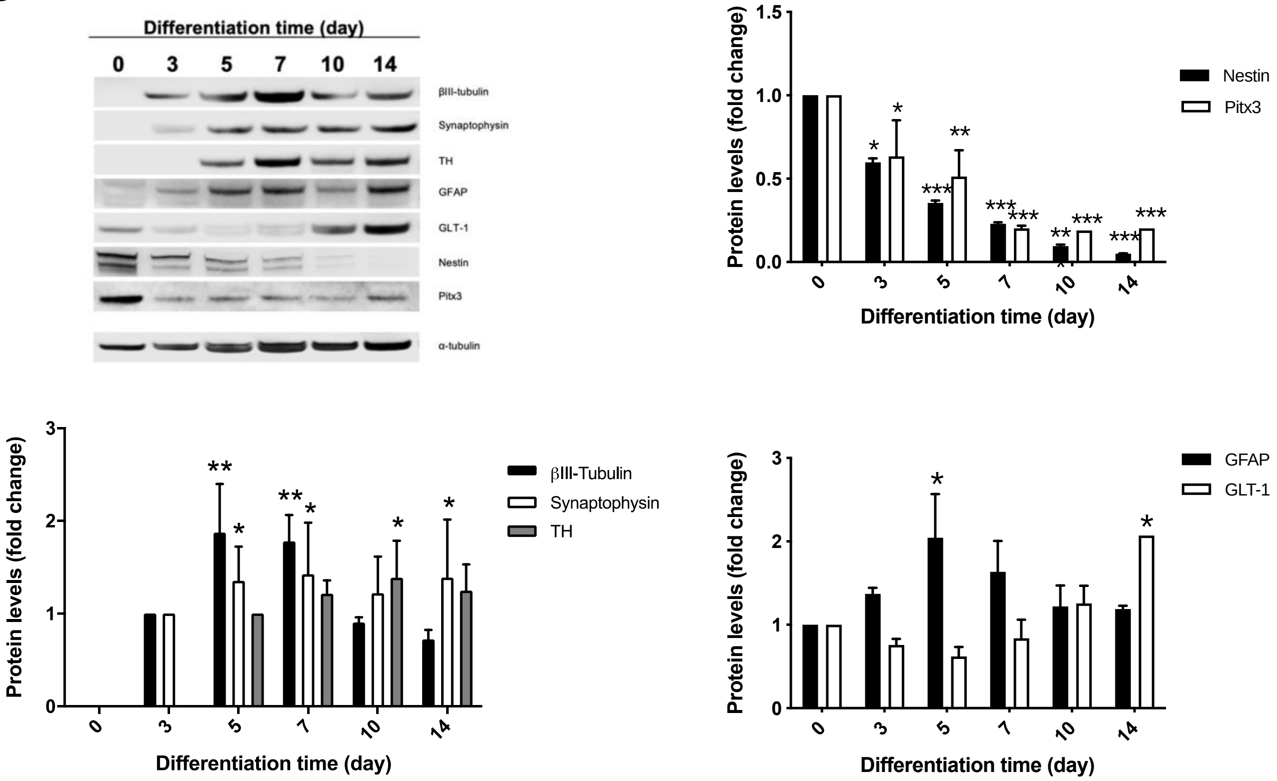

C

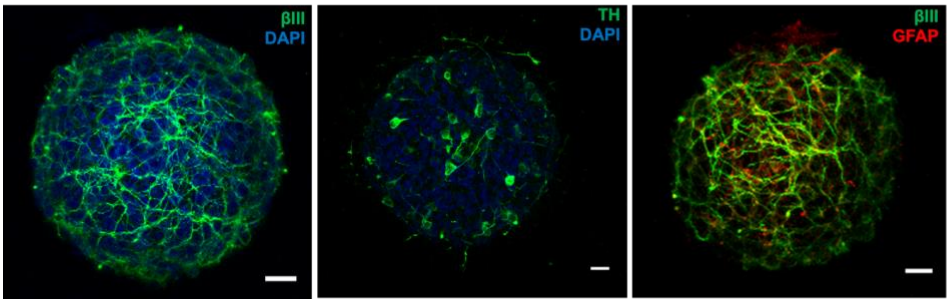

Figure S2. LUHMES neurospheroids culture neural population characterization. (**A**) RT-qPCR analysis of Nestin, βIII-tubulin, synaptophysin, TH and DAT gene expression; fold increase in gene expression of neurospheroids along culture time normalized for 0d of differentiation. Statistical differences are donated as ***p<0.001, **p<0.01 and *p<0.05 relative to 0d differentiation. (**B**) Detection of βIII-tubulin, synaptophysin, TH, glial fibrillary acidic protein (GFAP), glutamate transporter (GLT-1), pituitary homeobox-3 (Pitx3) and Nestin by Western blot analysis and corresponding fold change in protein levels obtained by densitometry normalized for 0d of differentiation; α-tubulin was used as loading control. Statistical differences are donated as *p<0.05, **p<0.01, ***p<0.001 relative to 0d differentiation, by two-way ANOVA analysis with Tukey's post multiple comparison test. (**C**) Immunofluorescence microscopy of neurospheroids (7d Diff); scale bar: 20 μm; βIII-tubulin (green), TH (green), GFAP (red), and DAPI (blue).

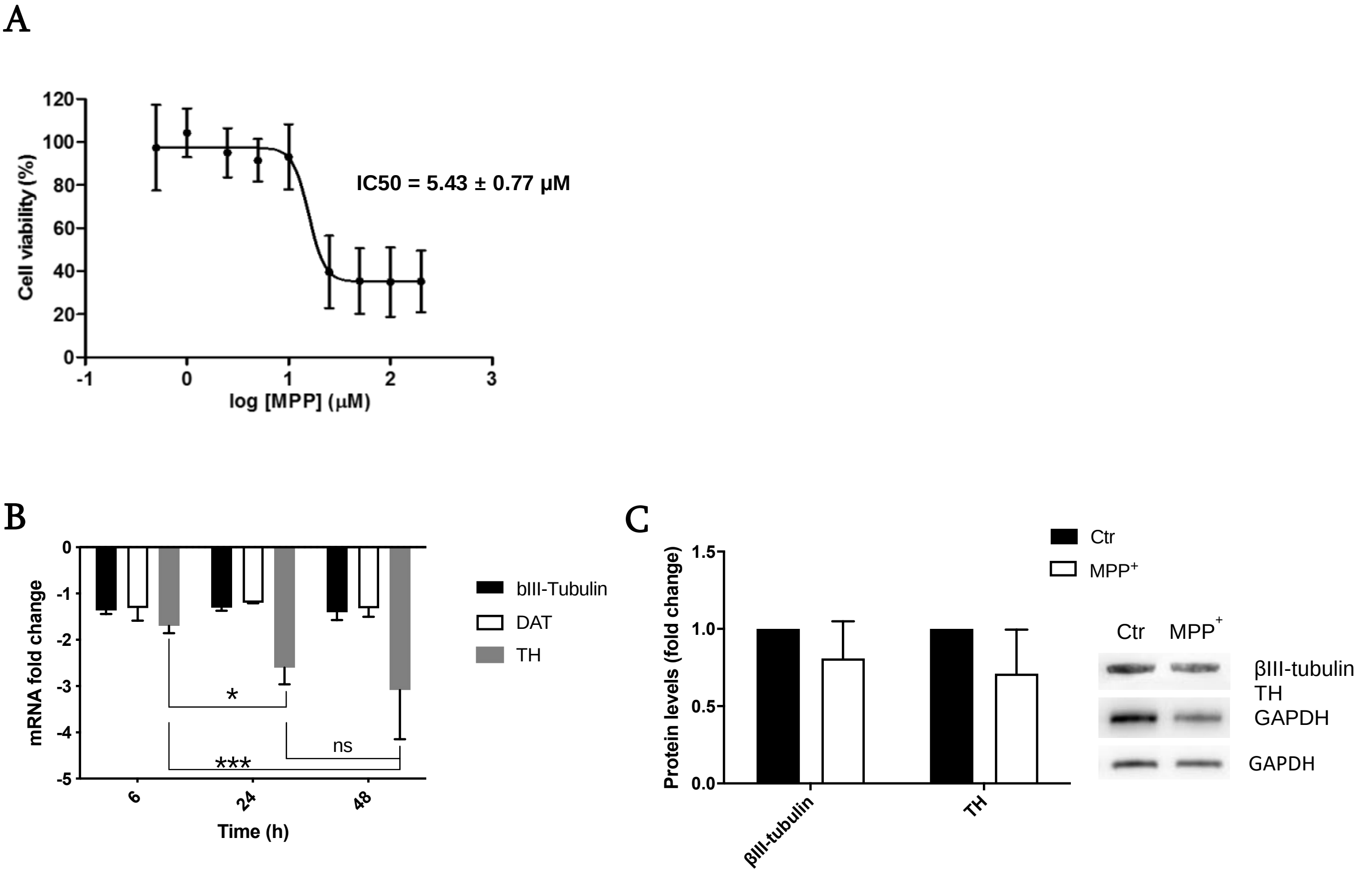

Figure S3. Differentiated LUHMES neurospheroids (7d Diff) were submitted to MPP$^+$. (**A**) Dose-response curve of 0.5-100 µM MPP$^+$ induced lesion for 24 hours with respective IC50, determined by Presto blue assay. (**B**) RT-qPCR analysis of βIII-tubulin, TH and DAT gene expression; fold change in gene expression of neurospheroids treated with 5 µM MPP$^+$ after 6h, 24h and 48h. Statistical differences are denoted as *p<0.05 or ***p<0.0001 as indicated, by two-way ANOVA analysis with Tukey's post multiple comparison test. (**C**) Detection of βIII-tubulin and TH by western blot analysis and corresponding fold change in protein levels obtained by densitometry and normalized for control condition (no MPP$^+$). Glyceraldehyde 3-phosphate dehydrogenase (GAPDH) was used as loading control. Data are mean ± SD of three independent cultures.

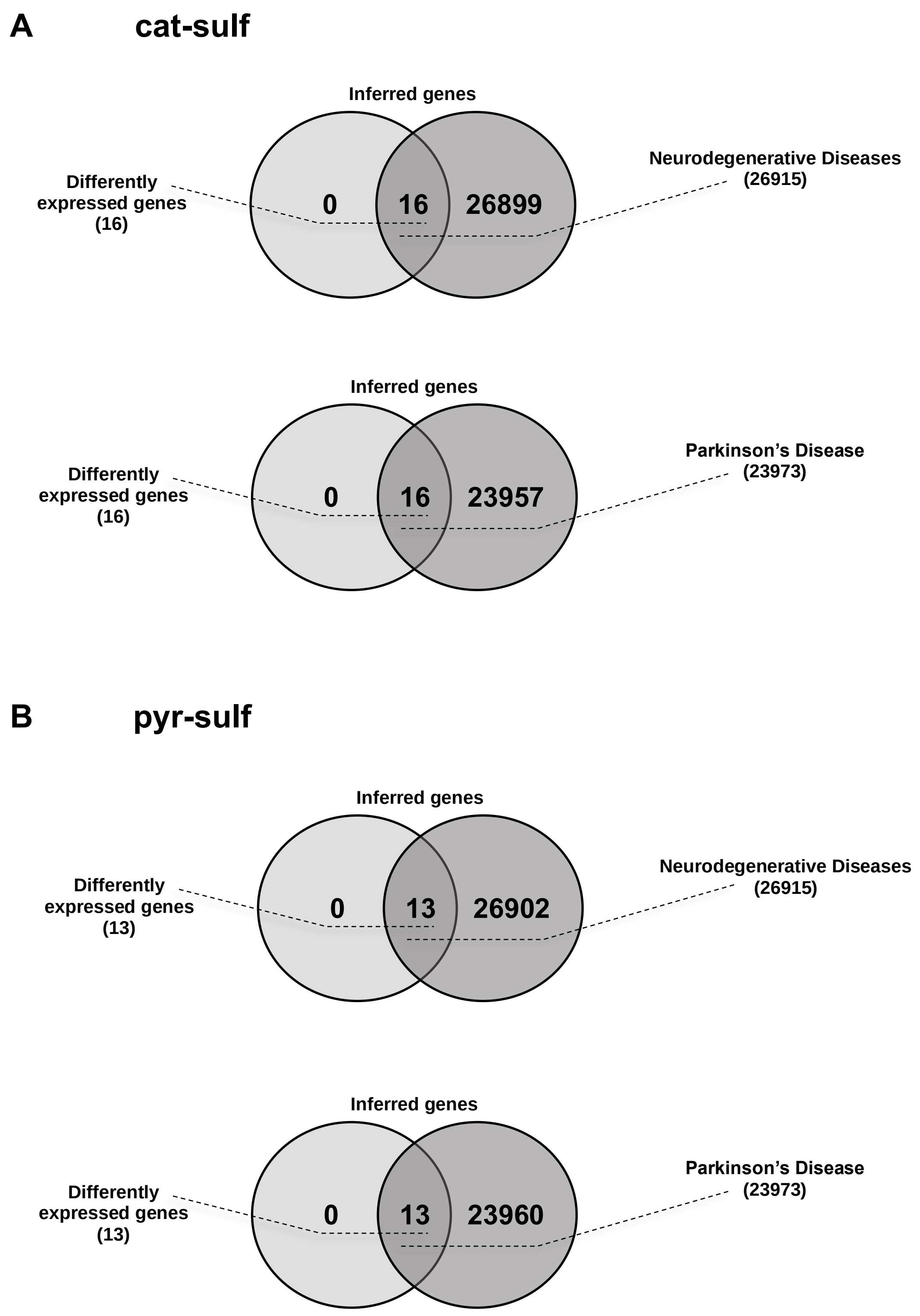

Figure S4. Venn diagram based on comparative toxicogenomic database showing the association of all the differentially expressed genes for cat-sulf (**A**) and pyr-sulf (**B**) treatments with neurodegenerative diseases and in particular with PD.

Supplementary references

[1] M. Serra, C. Correia, R. Malpique, C. Brito, J. Jensen, P. Bjorquist, M. J. T. Carrondo and P. M. Alves, *PLoS One* 2011, 6, e23212.

[2] S. Sá Santos, L. L. Fonseca, M. A. R. Monteiro, M. J. T. Carrondo and P. M. Alves, in *Journal of Neuroscience Research* **2005**, 79, 26.

[3] M. A. Gaffield and W. J. Betz, *Nat. Protoc.* **2006**, 1, 2916.

[4] A. P. Terrasso, C. Pinto, M. Serra, A. Filipe, S. Almeida, A. L. Ferreira, P. Pedroso, C. Brito and P. M. Alves, *J. Biotechnol.* **2015**, 205, 82.

[5] D. Simão, C. Pinto, S. Piersanti, A. Weston, C. J. Peddie, A. E. P. Bastos, V. Licursi, S. C. Schwarz, L. M. Collinson, S. Salinas, M. Serra, A. P. Teixeira, I. Saggio, P. A. Lima, E. J. Kremer, G. Schiavo, C. Brito and P. M. Alves, *Tissue Eng. - Part A* **2015**, 21, 654.
